# A continuum mechanics model of the plant cell wall reveals interplay between enzyme action and cell wall structure

**DOI:** 10.1140/epje/s10189-023-00396-2

**Published:** 2024-01-06

**Authors:** Euan T. Smithers, Galane J. Luo, Rosemary J. Dyson

**Affiliations:** 1 School of Mathematics, University of Birmingham, Birmingham, B15 2TT UK; 2 Sainsbury Laboratory, University of Cambridge, Bateman street, Cambridge, CB2 1LR Cambridgeshire UK

## Abstract

**Abstract:**

Plant cell growth is regulated through manipulation of the cell wall network, which consists of oriented cellulose microfibrils embedded within a ground matrix incorporating pectin and hemicellulose components. There remain many unknowns as to how this manipulation occurs. Experiments have shown that cellulose reorients in cell walls as the cell expands, while recent data suggest that growth is controlled by distinct collections of hemicellulose called biomechanical hotspots, which join the cellulose molecule together. The enzymes expansin and Cel12A have both been shown to induce growth of the cell wall; however, while Cel12A’s wall-loosening action leads to a reduction in the cell wall strength, expansin’s has been shown to increase the strength of the cell wall. In contrast, members of the XTH enzyme family hydrolyse hemicellulose but do not appear to cause wall creep. This experimentally observed behaviour still awaits a full explanation. We derive and analyse a mathematical model for the effective mechanical properties of the evolving cell wall network, incorporating cellulose microfibrils, which reorient with cell growth and are linked via biomechanical hotspots made up of regions of crosslinking hemicellulose. Assuming a visco-elastic response for the cell wall and using a continuum approach, we calculate the total stress resultant of the cell wall for a given overall growth rate. By changing appropriate parameters affecting breakage rate and viscous properties, we provide evidence for the biomechanical hotspot hypothesis and develop mechanistic understanding of the growth-inducing enzymes.

**Graphic Abstract:**

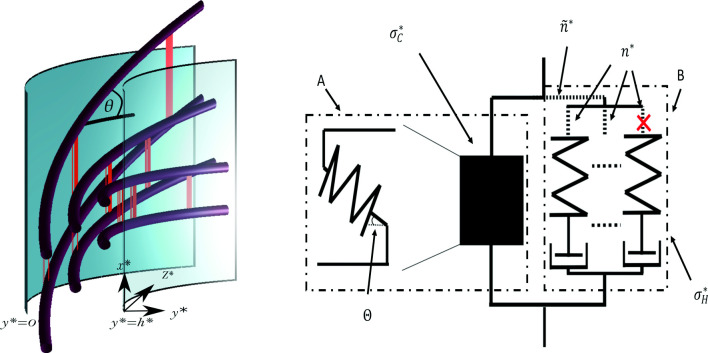

## Introduction

Faced with climate change and population growth, humanity needs plants that can cope with extreme weather events, diseases and rising demands on yield. As a result, understanding plant growth is essential to secure a sustainable future [1, 2]. Modification and adaptations to the plant genomes can provide a solution to optimising plant development. However, there remain many intriguing mysteries behind the mechanisms underlying plant growth.

As plants grow, their cell walls need to be strong enough to resist bursting but weak enough to allow permanent expansion. Controlled cell wall growth is an amazing feat, where some cells can increase in volume by over 30,000 times as they move from the meristem into maturation [3]. The driving force behind cell growth is the isotropic turgor pressure maintained by the uptake of water into the vacuole accompanied by an increase in cytoplasmic volume. This pressure inflates the cell membrane whose expansion is then restricted by the cell wall (a balloon in a box) creating a great tension in the cell wall, equivalent to 100–1000 atmospheres of tensile stress [3]. A stressed cell wall will deform elastically or plastically depending on the cell wall mechanical properties. Plastic deformation or growth begins when the mechanical load (turgor pressure) exceeds some critical value (yield threshold). This extension leads to thinning of the cell which can be balanced by the deposition of new wall material to maintain cell wall integrity. Since turgor acts in all directions, cell walls must be highly anisotropic to allow directional growth.

One of the earliest models of plant cell growth in one direction was the Lockhart equation, which states that the relative elongation rate, $\alpha ^*$, is proportional to the turgor pressure, $P^*$, if it is above a yield threshold, $Y^*$ [4]. This idea is expressed in the form:

1 $$\begin{aligned} \alpha ^*=\frac{1}{L^*}\frac{\text {d}L^*}{\text {d}t^*}= {\left\{ \begin{array}{ll} 0, \quad \quad \quad \quad \,\, \quad \ P^*\le Y^*, \\ \phi ^*(P^*-Y^*),\ \ P^*>Y^*, \end{array}\right. } \end{aligned}$$

where $L^*$ is the length of the cell and $\phi ^*$ is the extensibility. If the pressure does not exceed the yield threshold, the cell does not grow as the turgor cannot overcome the strength of the wall. In this article, we use the word “strength” to imply the cell wall’s ability to resist deformation. This formulation was a useful initial model, but our understanding of plant growth has now improved. Rather than changes in turgor pressure, growth is often controlled via careful manipulation of the cell wall, which results in changes to the yield threshold or the extensibility [5]. General reviews on mechanical modelling of plant growth can be found in [6–8], while further information on the biological details can be found in [5, 9–11].

In this paper, we create a mathematical model to investigate the interplay between cell wall structure and enzyme action in order to understand experimentally observed behaviour. Enzyme action is a crucial process in cell wall growth; however, it is generally under-researched in the biological context with even less modelling undertaken [6, 12].

The cell wall consists of three main components, cellulose (CMF), hemicellulose (e.g arabinoxylan or xyloglucan) and pectin [5, 9, 13–15]. The cell wall properties are carefully mediated via active control of the wall’s mechanical structure (e.g. by enzymatic action or new material deposition), altering either the yield or the post-yield behaviour and ultimately affecting the direction and rate of growth.

On the microscale, bond breakage and polymer network rearrangement (wall loosening) result in the relaxation of wall stress, allowing for the viscous flow of the cell wall. Cell wall loosening can be mediated by the action of proteins or enzymes, such as expansins, xyloglucan endotransglucosylase/hydrolase (XTH), and pectin-modifying enzymes, and are regulated by the action of hormones (morphogens), such as auxin, gibberellins, and abscisic acid [3, 10].

Cellulose are long and stiff molecules embedded in a matrix of hemicellulose and pectin. Cellulose are deposited in the cell wall in lamella layers at a variety of angles [16], typically perpendicular to the growth direction, circling the cell, where they reinforce the cell against radial expansion [9]. Cellulose molecules are also responsible for resisting the majority of the cell wall tension [17]. Experimental observations of the cell wall have found that cellulose molecules are transversely directed after deposition in the inner cell wall and reorient to a longitudinal direction as they move to the outside of the cell wall during growth [18–22], however this might not always occur [23].

How the rings of cellulose are connected is not precisely known [12]. It was previously thought that cellulose molecules are joined together via a tethered network where the cellulose fibres run parallel to each other and are continuously joined together by hemicellulose, which form hydrogen bonds with the cellulose and peel off when the network is deformed [10]. There are several problems with this theory. Simulations have revealed that assuming a tethered network structure results in a much weaker cell wall than experimentally observed [24]. It was also found that some plants that lack xyloglucan (mutant forms of Arabidopsis and celery) displayed only a small amount of growth reduction [14] implying the role of xyloglucan in cell wall strength could have been exaggerated. According to a finite element model featuring a network of cellulose molecules tethered together by hemicellulose via hydrogen bonds, a deformed network is not strong enough to withstand the strain caused by turgor [25]. These studies present evidence that the tethered network model is not a feasible explanation as to how the cell wall retains integrity. These results emphasise the mechanical role of other molecules like pectin and suggest that when xyloglucan is present, it could be concentrated around a limited number of distinct biomechanical hotspots (hotspots) where cellulose molecules come into close proximity with one another [14].

These concentrated hotspots could allow for controlled extension of the cell wall where these distinct spots are selected to allow slippage [9, 10, 14, 26]. Pectin is also theorised to have a role to play in these hotspots [16]. A model testing this theory considered a network of cellulose connected by hotspots represented as linear springs [27]. The model hypothesises that a group of short xyloglucan strands is stiffer than a single long strand, and when combined with pectin, the cell wall can produce the requisite wall stiffness to oppose turgor. The hotspot hypothesis claims that a small amount of degradation of the hotspots could lead to the load being carried by pectin, which then enables the viscous flow of the cell wall, providing a possible mechanism for growth.

Some enzymes affect the cell walls and possibly interact with the hotspots. Note that in this article, we refer to “wall loosening” as the action that directly causes stress relaxation, creep and hence growth, and “wall softening” as a decrease in the Young’s modulus [10]. XTH has been shown to have a hydrolysing action on the cell wall where it can cut and rejoin xyloglucans; despite this observed effect, it strangely does not induce significant cell wall extension [3, 10]. Cel12A, an enzyme present in fungi, has been shown to cause wall loosening. It has been suggested that Cel12A targets the hotspots by performing hydrolysis at these sites and leads to a reduction in wall strength [10, 28] and thus causes both wall loosening and wall softening [12]. Modelling efforts have offered an explanation of why these two enzymes hydrolysis action’s have different effects; using coarse-grained molecular dynamics it was found that cellulose is the main load-bearing component, which could be the reason why enzymes purely targeting xyloglucan are ineffective [17]. Expansin is a pH-controlled wall-loosening protein [3]. Some experiments have shown expansin action to induce growth [12, 29], while in contrast, other tests have observed the cell walls withstand more force without bursting [10]. Unlike Cel12A, Expansin action seems to loosen but not soften the wall [12], but the mechanism remains unknown. There has been no observed enzymatic action by expansin [30], so this effect could be due to force dissipation by $\alpha$-expansin. It is hypothesised that expansin targets the hotspots as there is evidence that they act on cellulose–cellulose sites [12, 26] where they may induce slippage of the fibres [30, 31].

This paper aims to produce a proof-of-concept model to test the plausibility of the hotspots hypothesis as an explanation for observed enzyme behaviour. We focus on inner tissues cells with predominately transversely orientated CMF with negligible cell division, for example cells within the root elongation zone [32–34]. We model the primary cell wall as a continuum incorporating crosslink dynamics (between cellulose and hemicellulose) and calculate the stress resultant when acted on by a prescribed growth rate. Using the model, we examine hypothesised expansin, Cel12A and XTH enzyme action, and investigate the consequences of cellulose reorientation. This model is designed to be a simplification of the system in order to test the feasibility behind proposed wall structure and protein mechanisms. The focus is therefore on the cellulose crosslinks and not on the pectin ground matrix, whose direct contribution (which was discussed in [35]) shall be neglected in the model, while its possible cellulose crosslinking will be incorporated. We begin with an explanation of the mathematical model of the cell wall in Sect. 2. This is followed by an analysis of the model outputs and a discussion of the implications of these findings in Sect. 4. We summarise the results and draw conclusions in Sect. 5.

## Model formulation

The model comprises three distinct aspects of cell wall dynamics: the emergent macroscopic stress and CMF orientation evolution from the microscopic cell wall network (Sect. 2.2.1), hotspot bond density evolution (Sect. 2.2.2) and enzyme action (Sect. 2.2.3). We will first detail the assumptions behind each aspect (Sect. 2.1), before deriving the relevant governing equations (Sect. 2.2). This model is based on the framework originally developed in [35], with significant differences in the treatment of material properties, network composition and fibre orientation evolution. We will simplify the governing equations through nondimensionalisation (Sect. 2.3) and solve the resulting system (Sect. 3). The solutions are further simplified via asymptotic reduction (Sect. 3.2) providing insights into the principal components controlling cell wall behaviour.

**Fig. 1:**
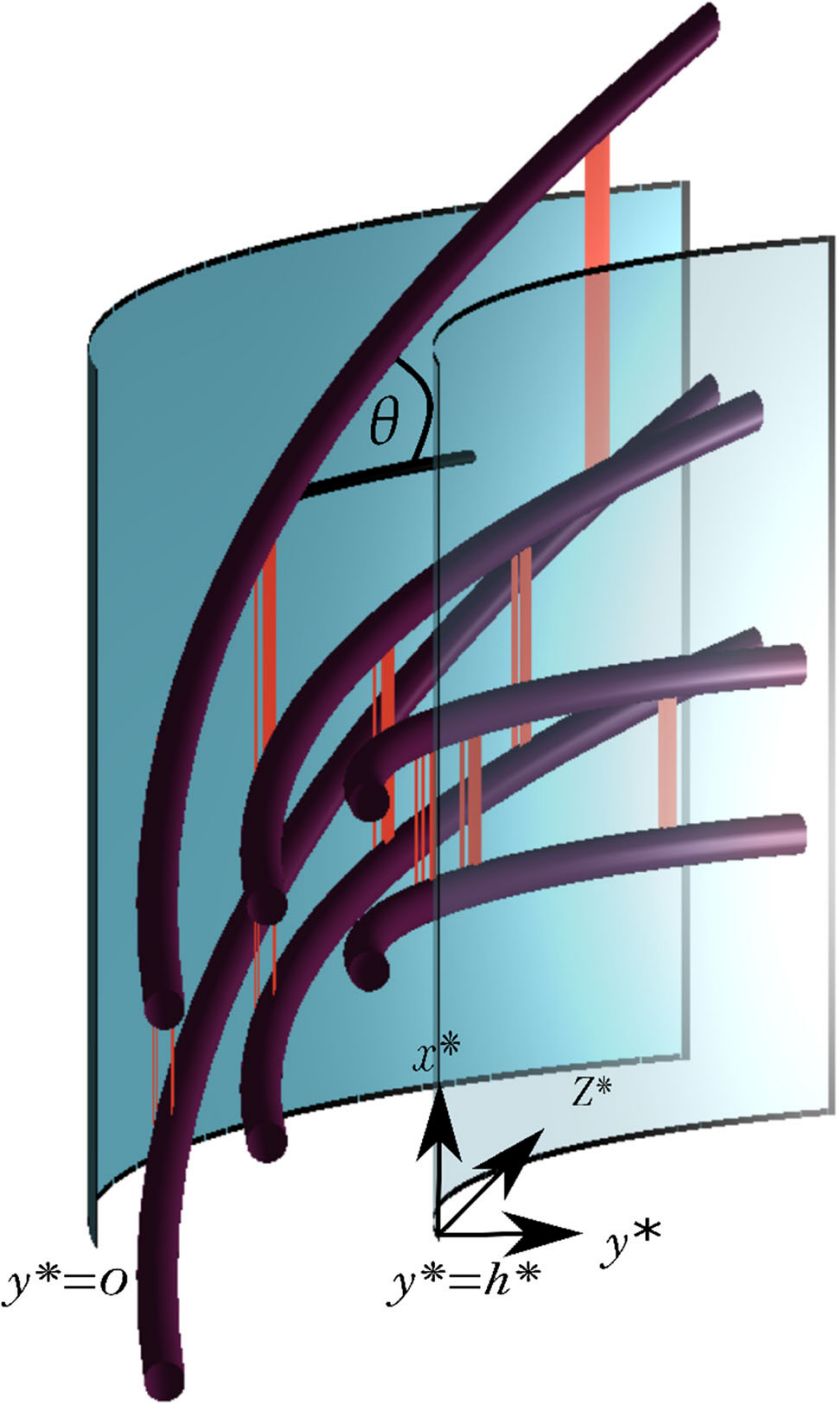
An idealised segment of the cell wall. The two curved (light blue) surfaces are the two boundaries of the cell wall. The thick (purple) rods represent the cellulose molecules reorienting as they approach the outside of the cell wall. The groups of thin (orange) fibres represent the hotspots which become increasingly stretched as they traverse to the outside of the cell. Equation (2) imposes this movement and deformation in the *x*-*y* plane. Note that the figure represents a simplification of biological reality as per the model, where biomechanical hotspots are distributed throughout the domain according to some dynamical density

### Model assumptions and set-up

The crucial output of the model is an expression for the axial stress resultant $\Sigma ^*$ of the cell wall (see Fig. 1). This stress resultant captures the strength of the cell wall generated by the underlying polymer network, as well as the relationship between turgor pressure and the growth rate; higher values of turgor increase the load on the cell wall, resulting in raised stress levels and possible further extension of the wall. The stress resultant is calculated by summing up the stress held by each component of the cell wall polymer network at all points in the cell wall. We assume that the cell wall is an evolving continuum with embedded cellulose molecules that are initially orientated perpendicularly to the axial direction. As the cell wall elongates, with growth rate $\alpha ^*$, all molecules are stretched, and the cellulose molecules are additionally reoriented. These cellulose molecules are connected by a mix of cell wall components including hotspots crosslinks as described in Sect. 1 where the hotspot bond density and the hemicellulose number functions are $n_{\text {hot}}^*$ and $n^*$, respectively. ($*$ denotes dimensional quantities throughout.) All functions that represent the whole hotspot are denoted with a subscript hot ( $_{\text {hot}}$ ) where functions that represent a single fibre are without. This bond density is dependent on the energy held in the fibres, meaning that as they get increasingly stretched via cell wall extension, they become more likely to break. As we are examining the principal growth direction, forces are resolved in this direction. Therefore, only *axial* hemicellulose are included since mechanically they matter the most. Enzyme action will be modelled by changes in the bond density evolution parameters. The wall segment is initially unstressed at time $t^*=0$ and undergoes uniform stretching in such a way that the cell wall grows with a fixed growth rate $\alpha ^*$. We now explain how this growth rate is imposed.

**Fig. 2:**
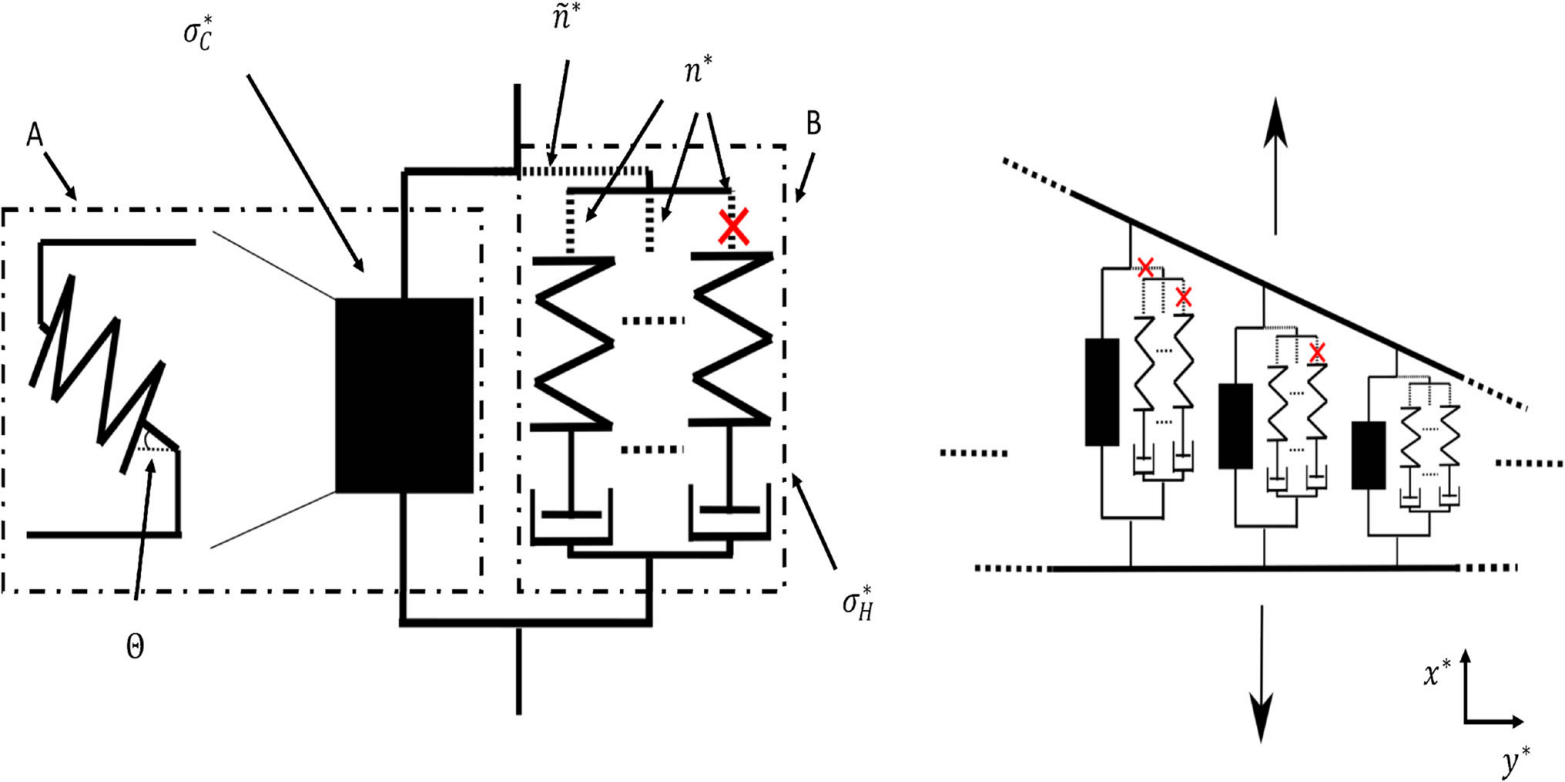
On the left a depiction of a single unit of the continuum model with the cellulose (box A) and hotspot (box B) contributions to the stress joined in parallel. The hotspot stress ${\sigma }^*_{\text {hot}}$ arises from a collection of hemicelluloses represented as Maxwell elements, consisting of a spring and viscous damper/dashpot, crosslinked with bond number $n^*$ (where the red cross represents a bond breakage). The cellulose stress $\sigma ^*_C$ is represented by a box that resists the strain elastically proportionally to the cellulose’s angle $\theta$. The whole unit’s stress is then dependant on the biomechanical hotspot density ${n}^*_{\text {hot}}$ such that when it is zero, the only contribution is coming from cellulose crosslinked by other molecules. On the right a representation of the cell wall continuum consisting of the single units being progressively stretched as they move through the cell wall causing the individual hemicelluloses bonds and hotspots to break (red crosses)

We introduce the coordinate system $(x^*,y^*,z^*)$ with the origin positioned on the edge of the outer wall (Fig. 1). The cell wall is stretched in the axial $x^*$ direction with the $y^*$, $z^*$ directions being perpendicular to the growth direction, such that $y^*$ points radially inwards, and $z^*$ tangential to the cell wall. We define $\theta$ to be the angle between the cellulose fibres and the $z^*$ axis. New wall material is deposited on the inner surface of the wall at $y^*=h^*$, moves through the wall at rate $\alpha ^*$ until it reaches the outer wall surface at $y^*=0$. We therefore model the growth via the incompressible flow field,

2 $$\begin{aligned} \textbf{u}^*=\alpha ^*(x^*,-y^*,0) \ \text {for} \ 0< y^*<h^*, \end{aligned}$$

meaning that $\text {d}x^*/\text {d}t^*=\alpha ^* x^*$ and $\text {d}y^*/\text {d}t^*=-\alpha ^* y^*$. This flow describes the material moving through the flat *x*-*y* plane in a thin segment of the cell wall (Fig. 1 and see figure 2 and 3 in [35] for further details). Due to the modelling domain being thin, we assume the cellulose molecules do not bend radially, and since there is no stretching in the $z^*$ direction, the model simplifies to a 2D system.

We now consider how a generic element of cell wall material changes in length as the cell wall stretches. Let $L^*(y^*,t^*)$ be the length of such a segment lying in the $x^*$ direction. The material extends with rate

3 $$\begin{aligned} \alpha ^*=\frac{1}{L^*}\frac{\partial L^*}{\partial t^*}, \end{aligned}$$

and so segment is therefore under an axial strain of $s^*=\frac{L^*}{L_0^*}-1$ where $L_0^*$ is the natural length of the material segment. If the initial length of a segment positioned at $y^*=y_i^*$ at the non-constant deposition time $t^*=\tau ^*$ is $L_i^*$, the segment length evolves according to $L^*=L_i^* e^{\alpha ^*(t^*-\tau ^*)}$ on the characteristic $y^*=y^*_i e^{-\alpha ^*(t^*-\tau ^*)}$ (from Eq. 3). The evolving segment length is then related to its position in the cell wall by $L^*=\frac{L_i^* y_i^*}{y^*}$, encoding both its extension and trajectory. As a result of this formulation, the system of equations described in Sect. 2.2 will all be partial differential equations dependent on both time and space.

To consider the amount of wall material contained in the wall segment and its deposition, we assume that at the inner surface of the cell wall, material is deposited such that constant cell wall thickness is maintained. This new wall material then gets pushed through the wall towards the outer surface via the flow (2). We find that wall density remains uniform for all time, assuming appropriate initial conditions (see appendix A for details). When new fibres are deposited, they are assumed to have zero stress and oriented in the *z* direction as they are yet to be subjected to tension or the material flow. This assumption will be reflected in the boundary conditions in Sect. 2.2.

### Principal equations

#### Stress resultant from the composite cell wall

The total stress resultant from the composite cell wall in the axial direction when growing in the axial direction depends on the mechanical properties of each constitutive (and evolving) part: the CMF which are bound together by both the hotspots and other cell wall components, the hemicellulose within the hotspots and the surrounding pectin ground matrix. We assume a standard linear solid-like system, so the total stress $\sigma ^*$ (taken from the stress components in the axial direction) is the sum of the stresses arising from the biomechanical hotspots $\sigma _{\text {hot}}^*$ and the cellulose molecules $\sigma _C^*$ (Fig. 2).

We let $\sigma _H^*$ be the stress held in a hemicellulose fibre which we assume to be characterised by a Maxwell element where the hemicellulose strain, $s_H$, is equal to the wall strain, $s=s_{H}$. The Maxwell assumption means that the hemicellulose strain is the sum of the elastic and viscous contributions (stretching a spring and viscous damper/dashpot in series), $s_{H}=s_{H,e}+s_{H,v}$, with the hemicellulose stress being equal to its elastic and viscous stress, $\sigma _H^*=\sigma _{H,e}^*=\sigma _{H,v}^*$. This implies that

4 $$\begin{aligned} \frac{1}{\nu _H^*}\frac{\partial \sigma _H^*}{\partial t^*}+\frac{1}{\mu ^*}\sigma _H^*=\frac{\partial s}{\partial t^*}, \end{aligned}$$

with the boundary and initial conditions of $\sigma ^*_H(h^*,\tau ^*)=\sigma ^*_H(y^*,0)=0$ where $\tau ^*$ is the time the fibre is deposited, $h^*$ the wall thickness, $\nu _H^*$ the hemicellulose Young’s modulus and $\mu ^*$ the material constant of viscosity. As each hotspot is a compact collection of hemicellulose fibres, we assume that the stress arising from the hotspots, $\sigma _{\text {hot}}^*$, is then the sum of the hemicellulose stresses contained within it, meaning $\sigma _{\text {hot}}^*=n^*\sigma _H^*$. Here it is assumed that the wall strain is uniformly distributed across all the fibres and that the deformation is smooth, i.e. the strain in the fibres is the same as the wall strain. It has been proposed that the strain distribution through the wall may be discontinuous, being smaller at the hotspots [11]. This effect could be incorporated through extension of the fibres, for example $s_{H}=\delta s$ or $s_{H}=s-\delta s_{C}$ where $s_{C}$ is the cellulose strain and $\delta <1$ and is some parameter to scale the amount of strain imposed onto the hotspots. This would, however, incorporate yet another unknown parameter while introducing little impact on the results. (It would scale the breakage rate down and the stress resultant up due to cellulose’s contribution.) Thus, discontinuity of the strain is not considered. Importantly, even though these hotspots are being strained the same amount, this strain is being released by the viscous slippage of the dashpots; so these fibres are under less elastic strain than the wall. And as the fibre breakage rate is solely dependent on this elastic strain (see Sect. 2.2.2), some aspects of the discontinuity are already included in the model.

Assuming the cellulose molecules are elastic, the stress term for the CMF in the axial direction is then,

5 $$\begin{aligned} \sigma ^*_C=\nu _C^* s_{C} \sin \theta , \end{aligned}$$

where $\nu _C^*$ is the Young’s modulus, $\theta$ the fibre angle, $\sin \theta$ a modifier that changes the cellulose stress as they become increasingly reoriented to the axial direction, and $s_{C}$ the strain of cellulose fibre, such that

6 $$\begin{aligned} s_C=\frac{L_C^*}{L_{C,0}^*}-1. \end{aligned}$$

where $L_{C,0}^*$ is the cellulose resting length.

The cellulose fibre angle $\theta$ and length $L_C^*$ are orientated and deformed by the flow $\textbf{u}^*$. Letting $\textbf{a}$ be the cellulose direction vector, we shall assume the cellulose network is uniform in the $x^*$ and $z^*$-direction as they are in the plane of the wall, and thus, $\textbf{a}=(\sin \theta ,0,\cos \theta )$. Assuming the cell wall is a composite material with a preferred direction under an incompressible, transversely isotropic viscous flow, the evolution of the fibre director field, $\textbf{a},$ can be described by [36],

7 $$\begin{aligned} \frac{\partial \textbf{a}}{\partial t^*}+ \left( \textbf{u}^*\cdot \nabla ^* \right) \textbf{a}+\zeta ^* \textbf{a}=(\textbf{a}\cdot \nabla ^*)\textbf{u}^* , \end{aligned}$$

where $\zeta ^*=\textbf{a}\cdot \textbf{e}^* \cdot \textbf{a}$ represents the strain rate in the direction of the matrix, with $\textbf{e}^*=\left( \nabla ^* \textbf{u}^*+\nabla ^* \textbf{u}^{*T}\right) /2$. We now have an expression that describes how cellulose fibres convect, stretch and reorient as they move through the cell wall via the flow, $\textbf{u}^*$. Equation (7) can be re-expressed in terms of $\theta$ using the definition of $\textbf{a}$ meaning $\zeta ^*=\alpha ^*\sin ^2\theta$, and upon substituting in Eq. (7) we obtain

8 $$\begin{aligned} \cos \theta \frac{\partial \theta }{\partial t^*}-\alpha ^* y^* \cos \theta \frac{\partial \theta }{\partial y^*}+\alpha ^*\sin ^3\theta =&\alpha ^*\sin \theta , \end{aligned}$$

9 $$\begin{aligned} -\sin \theta \frac{\partial \theta }{\partial t^*}-\alpha ^*y^*\sin \theta \frac{\partial \theta }{\partial y^*}+\alpha ^*\sin ^2\theta \cos \theta =&0 . \end{aligned}$$

Computing $\sin \theta \times (8) + \cos \theta \times$ (9) and dividing the result by $\sin \theta \cos \theta$ yield

10 $$\begin{aligned} \frac{\partial \theta }{\partial t^*}-\alpha ^*y^*\frac{\partial \theta }{\partial y^*}-\alpha ^*\sin \theta \cos \theta =0, \end{aligned}$$

with boundary and initial conditions, respectively, being $\theta (h^*,\tau ^*)=\theta (y^*,0)=\theta _0$ (note $\theta _0\ne 0$, else the solution is simply $\theta = 0$ for all $t^*>0$).

From the derivation of Eq. (7), we also have an expression that describes the evolution of the cellulose length $L_C^*$ when stretched via the flow as

11 $$\begin{aligned} \frac{1}{L_C^*}\frac{\text {d}L_C^*}{\text {d}t^*}&=\textbf{a}\cdot \left( \left( \textbf{a}\cdot \nabla ^*\right) \textbf{u}^*\right) =\alpha ^*\sin ^2 \theta , \end{aligned}$$

with boundary and initial conditions being $L_C^*(h^*,\tau ^*)=L_C^*(y^*,0)=L_{C,0}^*$.

Finally, the total stress resultant in the cell wall is then the sum of the stress of all components when crosslinked in each “layer” of the cell wall, i.e. integrating over the thickness of the wall, which gives

12 $$\begin{aligned} \Sigma ^*=\int ^{h^*}_0 \rho ^*(1+A^*n_{\text {hot}}^*) \sigma _C^*+n_{\text {hot}}^*\sigma _{\text {hot}}^* \ \text {d}y^*+\Gamma ^*\alpha ^*, \end{aligned}$$

where $\rho ^*$ is the density of cellulose and $(1+A^*n_{\text {hot}}^*)$ is the modification of the cellulose stress due to crosslinking, noting that cellulose can only contribute to the wall stress if they are connected to one another, otherwise they are just pulled apart.. The cellulose modification term in (12) has two contributions: the first represents non-hotspot crosslinks and the second hotspots crosslinks, with $A^*$ controlling the magnitude of the contribution. The integral is the contribution from the CMF (first term with $\sigma _C^*$) and the xyloglucan hotspots (second term with $\sigma _{\text {hot}}^*$), with the xyloglucan term dependent on the hotspot density for the same reasons as cellulose; the final term is the pectin matrix contribution which is assumed to provide an extensional viscosity due to its properties [37, 38], with $\Gamma ^*$ being the stiffness density of the matrix. This concludes the description of the stress resultant, so we proceed to characterise the crosslinking dynamics.

#### Bond density evolution equation

We now introduce equations that describe the hotspots density and hemicellulose number, $n_{\text {hot}}^*$ and $n^*$ respectively. Assuming that the CMF do not break before the hotspots, it is then the hotspots connections that rupture to allow slippage of the cell wall components. We assume that no new bonds are formed inside the cell wall. To address this potential limitation, we could have included a stochastic bond reformation term. However, this would be equivalent to uniformly decreasing all bond breakage rates, so it would introduce more unknown parameters without producing new effects on the results in Sect. 4. The hotspots and the hemicellulose fibres within them are advected through the cell wall according to a Smoluchowski equation [39], with both $n_{\text {hot}}^*$ and $n^*$ undergoing energy-dependant breakage:

13 $$\begin{aligned} \frac{\partial n_{\text {hot}}^*}{\partial t^*}-\alpha ^* y^* \frac{\partial n_{\text {hot}}^*}{\partial y^*}=&-k^*_{\text {off},\text {hot}} n_{\text {hot}}^* , \end{aligned}$$

14 $$\begin{aligned} \frac{\partial n^*}{d t^*}-\alpha ^* y^* \frac{\partial n^*}{\partial y^*}=&-k^*_{\text {off}} n^*, \end{aligned}$$

where $n_{\text {hot}}^*(h^*,\tau ^*)=n_{\text {hot}}^*(y^*,0)=n_{0,hot}^*$, and $n^*(h^*,\tau ^*)$ $=n^*(y^*,0)=n_0^*$, with the breakage rates defined as

15 $$\begin{aligned} k^*_{\text {off},\text {hot}}&=k_{0,\text {hot}}^*\exp \left( \frac{\hat{\beta }^2}{k_b^* T^*}U_{\text {hot}}^*(y,t)\right) , \end{aligned}$$

16 $$\begin{aligned} k^*_{\text {off}}&=k_{0}^*\exp \left( \frac{\gamma ^2 }{k_b^* T^*}U^*(y,t)\right) . \end{aligned}$$

In Eqs. (15) and (16), $k_b^* T^*$ is the thermal energy ($k_b^*$ being the Boltzmann’s constant and $T^*$ the absolute temperature), $k_{0,hot}^*$ and $k_{0}^*$ are the breakage rates when the fibres are unstressed, $U_{\text {hot}}^*$ and $U^*$ the deformation free energies and $\hat{\beta }$ and $\gamma$ are parameters controlling how strongly the energies affect the breakage rates. This formulation entails a direct relationship between the stress held in the fibres and the breakage rates and has been used successfully in other models of adhesion dynamics [40–43]. To ensure that the crosslinks stretch a significant length before breaking [35], we take $\hat{\beta }, \gamma \ll 1$; alternatively, taking larger values of these parameters allows us to model increased breakage rates caused by, for example, heightened hydrolysis action. As hemicellulose bonds break, the hotspots become easier to rupture, and as a result, we take $\hat{\beta }^2=(\frac{n_0^*}{n^*}-1)\beta ^2$. We also assume that the hotspots are stronger than the hemicellulose crosslinks, so $\beta <\gamma$.

To calculate the free energy potential in a single fibre, we consider the force $\textbf{F}^*$ on a fibre as it moves along the trajectory $\textbf{x}^*(t^*)=(x^*(t^*),y^*(t^*),z^*(t^*))$. We set $y^*(t)=z^*(t)=0$ as the molecules are only being extended in the axial direction, so

17 $$\begin{aligned} x^*(t^*)=s_{H,e}^*, \end{aligned}$$

with $s_{H,e}^*$ being the elastic extension. The work done by $\textbf{F}^*$ is then

18 $$\begin{aligned} W^*=\int ^{s_{H,e}^*}_0 F_x^* \ \text {d}x^*. \end{aligned}$$

Since the bond breakage is dependent on elastic strain, the force is also elastic, meaning $F_x^*=-\kappa ^* x^*$ where $\kappa ^*$ is the stiffness of the springs and is equal to the area multiplied by $\nu _H^*$ divided by the length of the cellulose molecules. Since temperature is constant, the free energy equals the potential/work done and so $U^*=\frac{\kappa ^*}{2}\left( s_{H,e}^*\right) ^2$. For the hotspot density potential, the force applied is $F_x^*=-\kappa _{\text {hot}}^* x^*$ for some stiffness $\kappa _{\text {hot}}^*$, which should be affected by the number of intact fibres in the hotspot, $n^*$; we therefore take $\kappa _{\text {hot}}^*=n^*\kappa ^*$, as the springs are in parallel so stiffness is additive and hence $F_x^*=-\kappa ^* n^*(y^*,t^*) x^*$. Then it follows that

19 $$\begin{aligned} W^* = \int ^{s_{H,e}^*}_0 F_x^* \ \text {d}x^*=-\frac{\kappa ^*}{2} n^*(y^*,t^*) (s_{H,e}^*)^2, \end{aligned}$$

and $$U_{\text {hot}}^*=\frac{\kappa ^*}{2} n^*(y^*,t^*) (s_{H,e}^*)^2$$; therefore,

20 $$\begin{aligned} k^*_{\text {off},hot}=k_{0,hot}\exp \left( \frac{\kappa ^*\beta ^2n_0^*\left( 1-\frac{n^*}{n_0^*}\right) }{2k_b^* T^*} (s_{H,e}^*)^2\right) . \end{aligned}$$

This completes the description of crosslinking, and we proceed to detail the implementation of enzyme action.

#### Enzyme action

We consider a simple model of enzyme action. Recall that the enzymes Cel12A and XTH perform hydrolysis/cutting action, where Cel12A targets the hotspots junctions (cutting both cellulose and hemicellulose) and XTH targets the hemicellulose fibres. We simulate the Cel12A and XTH actions by increasing $\beta$ and $\gamma$, respectively, in Eqs. (15) and (16). As Cel12A also digests the cellulose molecules, it might also decrease the cellulose density $\rho$. As the focus is on the hotspots, the modelling of this effect will be left to the appendix E.

Due to the lack of consensus on how expansin works, we try two different simple methods of enzyme action. As mentioned in Sect. 1, expansin may work by allowing slippage in the fibres [31]. The first method entails decreasing the viscosity of the dashpots, i.e. decreasing their resistance to the flow. This effect is modelled by the equation

21 $$\begin{aligned} \frac{\partial \mu ^*}{\partial t^*}=E^*\mu ^* \left( 1-\frac{\mu ^*}{\mu _1^*} \right) , \end{aligned}$$

with the conditions $\mu ^*(h^*,\tau ^*)=\mu ^*(y^*,0)=\mu _0^*$, where $E^*$ is the expansin action rate, $\mu _0^*$ the initial viscosity and $\mu _1^*$ the target viscosity with $\mu _1^*<\mu _0^*$. We choose this form to keep the model as simple as possible while ensuring that $\mu ^*$ decreases and that $\mu ^*\ne 0$.

The second method imposes expansin action by increasing the resting length of the springs in the system allowing for stress relaxation. We assume that the resting length growth rate depends on the strain of the spring, rather than the extension; this assumption avoids the resting length exceeding the actual length. Thus,

22 $$\begin{aligned} \frac{\partial L_0^*}{\partial t^*}=E^*\left( \frac{L^*}{L_0^*}-1\right) , \end{aligned}$$

with the boundary and initial conditions, $L_0(h^*,\tau ^*)=L_0^*(y^*,0)=l_0^*$. With the system of equations fully described, we now proceed to simplify them through nondimensionalisation.

### Nondimensionalisation

We nondimensionlise the system according to

23 $$\begin{aligned}&t^*=\frac{t}{k_{0,hot}^*}, \ \tau ^*=\frac{t}{k_{0,hot}^*}, \ n_{\text {hot}}^*=n_{0,hot}^* n_{\text {hot}}, \nonumber \\&n^*=n_0^*n, \ y^*=h^*y, \ y^*_i=h^*y_i, \nonumber \\&L^*=L_0^* L,\ L^*_C=L_{C,0}^*L_C, \ \Sigma ^*=\mathcal {E}^* \Sigma , \nonumber \\&\alpha ^*=k_{0,hot}^* \alpha , \ k^*_{\text {off},hot}=k_{0,hot}^* k_{\text {off},hot}, \ \nonumber \\&k^*_{\text {off}}=k_{0,hot}^*k_{\text {off}}, \ \sigma ^*_{H}=\nu _H^* \sigma _{H},\nonumber \\&\sigma _C^*=\nu _C^* \sigma _C, \ L_0^*=l_0^* L_0,\nonumber \\&\Gamma ^*=\frac{\mathcal {E}^* \Gamma }{ k_{0,hot}^*}, \ \mu ^*=\mu _0^*\mu , \nonumber \\&\text {and} \ E^*=k_{0,hot}^* E \ (\text {when using the mechanism}\nonumber \\&\qquad \text {described in Eq.} (21)) \nonumber \\&\text {or} \ \ E^*=k_{0,hot}^* l_0^* E \ (\text {for the description in Eq.} (22)), \end{aligned}$$

where we define $\mathcal {E}^*=\nu _C^* h^* \rho ^*$. Equations (3)–(22) are simplified as follows and are later solved in Sect. 3. The wall length Eq. (3) becomes,

24 $$\begin{aligned} \frac{\partial L}{\partial t}=\alpha L, \end{aligned}$$

with $L(1,\tau )=L(y,0)=1$. For the hemicellulose stress Eq. (4), after nondimensionalisation we have,

25 $$\begin{aligned} \frac{\partial \sigma _H}{\partial t}+\omega \sigma _H=\frac{\partial s}{\partial t}, \end{aligned}$$

with $\sigma _H(1,\tau )=\sigma _H(y,0)=0$, where $\omega =\frac{\nu _H^*}{\mu _0^*\mu k_{0,hot} }$ and $s=\frac{L}{L_0}-1$ (where $L_0=1$ when expansin is not acting upon it). The cellulose stress Eq. (5) is then simply

26 $$\begin{aligned} \sigma _C= s_C \sin \theta , \end{aligned}$$

where $s_C=L_C-1$. The length of the cellulose fibres Eq. (11) is then

27 $$\begin{aligned} \frac{1}{L_C}\frac{\partial L_C}{\partial t}=\alpha \sin ^2 \theta \end{aligned}$$

where $L_C(1,\tau )=L_C(y,0)=1$. Equation (10) describing the cellulose angle becomes:

28 $$\begin{aligned} \frac{\partial \theta }{\partial t}-\alpha y\frac{\partial \theta }{\partial y}-\alpha \sin \theta \cos \theta =0, \end{aligned}$$

with $\theta (1,\tau )=\theta (y,0)=\theta _0$. The evolving hotspots bond density and hemicellulose bond number Eqs. (13) and (14) are now

29 $$\begin{aligned}&\frac{\partial n_{\text {hot}}}{\partial t}-\alpha y \frac{\partial n_{\text {hot}} }{\partial y}=-k_{\text {off},hot} n_{\text {hot}} \nonumber \\&\text {where} \ k_{\text {off},hot}=\exp \left( \beta _{\text {hot}}^2 \varsigma _{\text {hot}} (1-n)s_{H,e}^{2}\right) , \end{aligned}$$

30 $$\begin{aligned}&\frac{\partial n}{\partial t}-\alpha y \frac{\partial n }{\partial y}=- k_{\text {off}} n \ \text {where} \ k_{\text {off}}=\breve{k}_0\exp \left( \gamma ^2 \varsigma s_{H,e}^{2}\right) , \end{aligned}$$

with $n_{\text {hot}}(1,\tau )=n_{\text {hot}}(y,0)=1$, $n(1,\tau )=n(y,0)=1$, $\beta _{\text {hot}}=\sqrt{n_0^*}\beta$, $\sigma _H=s_{H,e}$, $\breve{k}_0=\frac{k_{0}^*}{k_{0,hot}^*}$ and $\varsigma _{\text {hot}}=\varsigma =\frac{ \kappa ^* L_0^{*2}}{2k_b^* T^*}$. Enzyme action on the viscosity on the dashpot (21) is now

31 $$\begin{aligned} \frac{\partial \mu }{\partial t}=E\mu \left( 1-\mathcal {M}\mu \right) \end{aligned}$$

with $\mu (1,\tau )=\mu (y,0)=1$ where $\mathcal {M}=\frac{\mu _0}{\mu _1}$. The enzyme action on the resting length (22) becomes:

32 $$\begin{aligned} \frac{\partial L_0}{\partial t}=E\left( \frac{L}{L_0}-1\right) \end{aligned}$$

with $$L_0(1,\tau )=L_0(0,y)=1$$.

Finally, the stress resultant Eq. (12) is now

33 $$\begin{aligned} \Sigma =\int ^1_0(1+a_1 n_{\text {hot}}) \sigma _C+a_2 n n_{\text {hot}}\sigma _{H} \ \text {d}y +\Gamma \alpha , \end{aligned}$$

where $a_1=A^*n_{0,hot}^*$ is the modification of the cellulose stress due to hotspot crosslinking, and $a_2=\frac{n_0^*n_{0,hot}^*\nu _H^*}{\rho ^* \nu _C^*}$ is the ratio of the cellulose and hemicellulose stiffness densities. We therefore have a closed system of 10 Eqs. (24)–(33) with a total of 10 unknowns, $\Sigma$, $n_{\text {hot}}$, *n*, $\sigma _C$, $\sigma _{\text {hot}}$, $L_0$, $\mu$, *s*, $s_C$ and $\theta$. In the next section, we will solve the system of equations.

## Model analysis

To evaluate the effect of the hotspots and enzyme action on the stress resultant and hence the overall mechanical behaviour, we proceed to analyse the model as described in Sect. 2.2. The equations may be solved via a (lengthy) semi-analytic method; we merely state the solutions in Sect. 3.1 and leave full details to the Appendices B, C and D. Since breakage rates are assumed to be small, we also employ asymptotic expansions to determine the leading-order behaviour of the cell wall, as shown in Sect. 3.2; as before full details are consigned to F for brevity.

### Semi-analytical solutions

Throughout the analysis, we recognise two contrasting regions of cell wall material: the thinning region $0\le y \le e^{-\alpha t}$ contains material already present at $t=0$, while the expanding $e^{-\alpha t} < y \le 1$ region contains newly-deposited material which has been added to the $y=1$ surface at some $t > 0$. Recall from Sect. 2.1 that due to the fluid flow, fibres deposited at time $t=\tau$ (which is non-constant) lie on a characteristic $y=y_i e^{-\alpha \bar{t}}$, where $\bar{t}=t-\tau$. For the initially present material, the deposition time is $\tau =0$ and initial position is $0 \le y_i \le 1$ meaning the extension is described by $L=y_i e^{\alpha t}$. For the later-deposited material, we have $\tau >0$ and $y_i=1$, and thus, the extension can be given in terms of its position in the wall as $L=\frac{1}{y}$.

Beginning with the fibre stress terms, in the absence of enzymatic action, $L_0$ and $\mu$ are constant, and hence, Eq. (25) may be solved using an integrating factor to find the axial hemicellulose stress,

34 $$\begin{aligned} \sigma _{H}=\frac{\alpha }{\alpha +\omega }\left( L-L^{-\frac{\omega }{\alpha }}\right) . \end{aligned}$$

In contrast, when enzyme action is incorporated, $L_0$ and $\mu$ are no longer constant, and hence, the solution (34) is invalid. To solve for variable spring rest length, we first divide Eq. (32) by $\frac{\text {d}y}{\text {d}t}=-\alpha y$ to reformulate the differential equation for $L_0$ in terms of *y*. This equation does not have an analytical solution, and hence, we proceed to derive a (forward) finite difference expression for $L_0$, which can be used for solving Eq. (25) with an integrating factor, yielding

35 $$\begin{aligned} \sigma _H=L^{-\frac{\omega }{\alpha }}\int ^{\bar{t}}_0 L^{\frac{\omega }{\alpha }} \left( \frac{\alpha L}{L_0(L)}-\frac{L}{L_0^2(L)}\frac{\text {d}L_0(L)}{\text {d}\hat{t}}\right) \ \text {d}\hat{t}, \end{aligned}$$

where $L_0$ is treated as a function of *L*.

For expansin action on the viscosity, $\mu$, we begin by solving Eq. (31) using separation of variables to get

36 $$\begin{aligned} \mu =\frac{1}{\mathcal {M}+(1-\mathcal {M})e^{-Et}} \end{aligned}$$

which means that $\omega =\omega _0\left( \mathcal {M}+(1-\mathcal {M})e^{-Et}\right)$ where $\omega _0=\frac{\nu _H^*}{\mu _0^*k_{0,hot}^*}$. Equation (25) is then solved to give

37 $$\begin{aligned} \sigma _H&=y^{\frac{\omega _0\mathcal {M}}{\alpha }}\exp \left( \frac{\omega _0}{E}(1-\mathcal {M})y^{\frac{E}{\alpha }}\right) \nonumber \\&\quad \times \int ^1_y \alpha y^{-2-\frac{\omega _0\mathcal {M}}{\alpha }}\exp \left( -\frac{\omega _0}{E}(1-\mathcal {M})y^{\frac{E}{\alpha }}\right) \ \text {d}\hat{t}, \end{aligned}$$

To determine the evolving bond densities and number, we solve (30) for *n* using the method of characteristics and combining Eqs. (24) and (C18) yields

38 $$\begin{aligned}&n=\exp \left( -\frac{\breve{k}_0}{\alpha }G\left( \frac{1}{L}\right) \right) \end{aligned}$$

39 $$\begin{aligned}&\text {with} \ G(y)\nonumber \\&\quad =\int ^{1}_y \frac{\exp (\gamma ^2 \varsigma s_{H,e}^2)}{\hat{y}} \ \text {d}\hat{y}. \end{aligned}$$

Similarly from Eq. (29),

40 $$\begin{aligned}&n_{\text {hot}}=\exp \left( -\frac{1}{\alpha }G_{\text {hot}}\left( \frac{1}{L}\right) \right) \end{aligned}$$

41 $$\begin{aligned}&\text {with}\ G_{\text {hot}}(y)\nonumber \\  &\quad =\int ^{1}_y \frac{\exp \left( \beta _{\text {hot}}^2 \varsigma _{\text {hot}} \left( 1- \exp \left( -\frac{\breve{k}_0}{\alpha }G(\hat{y})\right) \right) s_{H,e}^2\right) }{\hat{y}} \ \text {d}\hat{y}. \end{aligned}$$

Solving Eq. (28) gives the cellulose angle from the horizontal,

42 $$\begin{aligned} \theta =\arctan \left( \tan \theta _0 L\right) , \end{aligned}$$

and thus, the cellulose extension length is,

43 $$\begin{aligned} L_C=\exp \left( \alpha \int ^{\bar{t}}_0 \sin ^2\theta (\hat{t}) \ \text {d}\hat{t} \right) . \end{aligned}$$

The stress resultant is hence constructed by separating the domain into two regions $\epsilon \le y \le e^{-\alpha t}$ and $e^{-\alpha t}<y\le 1$, to give

44 $$\begin{aligned}&\Sigma = \left( e^{-\alpha t}- \epsilon \right) \left( \left( 1+a_1\exp \left( -\frac{1}{\alpha }G_{\text {hot}} \left( e^{-\alpha t}\right) \right) \right) \right. \nonumber \\&\quad \left. \times \left( \exp \left( \alpha \int ^t_0 \sin ^2\theta (\hat{t}) \text {d}\hat{t}\right) -1\right) \right. \nonumber \\&\qquad \sin \left( \arctan \left( \tan (\theta _0)e^{\alpha t}\right) \right) \nonumber \\&\quad +a_2\exp \left( -\frac{k_0}{k_{0,hot}}\frac{1}{\alpha }G\left( e^{-\alpha t}\right) \right) \nonumber \\&\qquad \left. \exp \left( -\frac{1}{\alpha }G_{\text {hot}}\left( e^{-\alpha t}\right) \right) \sigma _H(t)\right) \nonumber \\&\quad +\int ^1_{e^{-\alpha t}}\left( 1+a_1\exp \left( -\frac{1}{\alpha }G_{\text {hot}}\left( y\right) \right) \right) \nonumber \\&\quad \left( \exp \left( \int ^1_y \frac{\sin ^2\theta (\hat{y})}{y} \text {d}\hat{y}\right) -1\right) \nonumber \\&\quad \times \sin \left( \arctan \left( \frac{\tan (\theta _0)}{y}\right) \right) \nonumber \\&\quad +a_2\exp \left( -\frac{k_0}{k_{0,hot}}\frac{1}{\alpha }G\left( y\right) \right) \nonumber \\&\quad \exp \left( -\frac{1}{\alpha }G_{\text {hot}}\left( y\right) \right) \sigma _H\left( y\right) \ \text {d}y +\Gamma \alpha . \end{aligned}$$

where $\sigma _H$ is given by Eqs. (34), (35) or (37) for the different expansin mechanisms. The small parameter $\epsilon$ denotes a cut-off for the outer region of the cell wall; this ensures that the cellulose stress does not go to infinity and that the molecules will eventually break rather than becoming infinitely long. The steady-state stress resultant as $t\rightarrow \infty$ is

45 $$\begin{aligned} \Sigma ^{\infty }&=\int ^1_{\epsilon }\left( 1+a_1\exp \left( -\frac{1}{\alpha }G_{\text {hot}} \left( y\right) \right) \right) \nonumber \\&\left( \exp \left( \int ^1_y \frac{\sin ^2\theta (\hat{y})}{y} \text {d}\hat{y}\right) -1\right) \nonumber \\&\times \sin \left( \arctan \left( \frac{\tan (\theta _0)}{y}\right) \right) \nonumber \\&+a_2\exp \left( -\frac{k_0}{k_{0,hot}}\frac{1}{\alpha }G\left( y\right) \right) \nonumber \\&\exp \left( -\frac{1}{\alpha }G_{\text {hot}}\left( y\right) \right) \sigma _H(y) \ \text {d}y +\Gamma \alpha . \end{aligned}$$

where the contributions from the fibres present at $t=0$ have now disappeared.

### Leading-order cell wall behaviour

An asymptotic expansion simplifies the model and extracts the leading-order component of the stress resultant integral when expansin is neglected (i.e. taking $\sigma _H$ from Eq. 34). The reader can skip this section or directly go to Eq. (57) for the final form. Since both $\gamma , \ \beta \ll 1$ by definition, we can expand the steady-state solution (45).

We first expand the integral *G* (Eq. 38) considering the respective regions $\gamma \ll y \le 1$ and $\epsilon \le y \ll \gamma$. By bounding the integral for *G*, integrating by parts and neglecting higher-order terms, we find

46 $$\begin{aligned} G(y)\approx {\left\{ \begin{array}{ll} \frac{y^2}{2\breve{\alpha }^2 \gamma ^2}\exp \left( \breve{\alpha }^2\frac{\gamma ^2}{y^2}\right) , \ \epsilon \le y \ll \gamma \\ \ln \left( \frac{1}{y}\right) , \ \hspace{17mm} \gamma \ll y \le 1, \end{array}\right. } \end{aligned}$$

where $\breve{\alpha }=\frac{\alpha }{\alpha +\omega }$ for notational simplicity. From Eq. (38), the asymptotic approximation of *n* is then,

47 $$\begin{aligned} n= {\left\{ \begin{array}{ll} \exp \left( -\frac{\breve{k}_0}{\alpha }\frac{y^2}{2\breve{\alpha }^2\gamma ^2}e^{\breve{\alpha }^2\frac{\gamma ^2}{y^2}}\right) , \quad \epsilon <y \ll \gamma \\ y^{\frac{\breve{k}_0}{\alpha }}, \hspace{34mm} \gamma \ll y \le 1. \end{array}\right. } \end{aligned}$$

The expansion of $G_{\text {hot}}$ proceeds similarly to give

48 $$\begin{aligned} G_{\text {hot}}(y)\approx {\left\{ \begin{array}{ll} \frac{y^2}{2 \breve{\alpha }^2\beta _{\text {hot}}^2}\exp \left( \breve{\alpha }^2\frac{\beta _{\text {hot}}^2}{y^2}\right) ,\ \epsilon <y \ll \beta _{\text {hot}} \\ \ln \left( \frac{1}{y}\right) , \ \hspace{17mm} \beta _{\text {hot}} \ll y \le 1. \end{array}\right. } \end{aligned}$$

Combining, we find

49 $$\begin{aligned} n_{\text {hot}}= {\left\{ \begin{array}{ll} \exp \left( -\frac{1}{\alpha }\frac{y^2}{2\breve{\alpha }^2\beta _{\text {hot}}^2}e^{\breve{\alpha } \frac{\beta _{\text {hot}}^2}{y^2}}\right) , \quad \epsilon <y \ll \beta _{\text {hot}} \\ y^{\frac{1}{\alpha }}, \hspace{31mm}\beta _{\text {hot}} \ll y \le 1 . \end{array}\right. } \end{aligned}$$

Notice that the simplification of $n_{\text {hot}}$ is not dependant on *n*. To complete the approximations for all *y*, we find switch-over values where the asymptotic behaviour of *n* and $n_{\text {hot}}$ changes from one regime to another (denoted $\chi$ and $\chi _{\text {hot}}$ for the hemicellulose and hotspots bond densities respectively). These are the *y*-values close to $\gamma$ or $\beta _{\text {hot}}$ such that the derivatives of the dominant exponents in *n* and $n_{\text {hot}}$ exceed some threshold *Q*, causing *n* and $n_{\text {hot}}$ to rapidly decrease. Thus,

50 $$\begin{aligned} \chi =\sqrt{\frac{\gamma ^2\breve{\alpha }^2}{\ln \left( \frac{\alpha }{\breve{k}_0} Q\right) }} \ \text {and} \ \chi _{\text {hot}}=\sqrt{\frac{\beta _{\text {hot}}^2\breve{\alpha }^2}{\ln \left( \alpha Q\right) }}. \end{aligned}$$

Approximations of the trigonometric term describing how much cellulose contribute to the axial stress and the cellulose extension are also required. To balance simplicity and accuracy, the following expansions are taken to such an order that they achieve a mean-squared error of $\approx 10^{-3}$ with respect to the numerical solution, and the domain is split into three regions corresponding to $\frac{\tan \theta _0}{y} > rapprox ,\approx ,\lessapprox 1$. In the first region, $\tan \theta _0+\delta <y\le 1$, where $\delta$ is a small number of our choosing to optimise the expansion’s overall accuracy, we Taylor expand $\sin$ and $\arctan$ in Eq. (45), where $\theta$ is described by Eq. (42). In the second and third regions, where $\frac{\tan \theta _0}{2}<y\le \tan \theta _0+\delta$ and $\epsilon \le y \le \frac{\tan \theta _0}{2}$, we use the expression $\sin \left( \arctan \left( \tan \theta _0\frac{1}{y}\right) \right) =\frac{\tan \theta _0}{\sqrt{\tan ^2\theta _0+y^2}}$ and Taylor expand around $y=\tan \theta _0$ and $y=0$, respectively. Hence,

51 $$\begin{aligned} \sin&\left( \arctan \left( \tan (\theta _0)\frac{1}{y}\right) \right) \nonumber \\&={\left\{ \begin{array}{ll} \frac{\tan \theta _0}{y}-\frac{1}{2}\frac{\tan ^3\theta _0}{y^3} +\frac{3}{8}\frac{\tan ^5\theta _0}{y^5}-\frac{5}{16}\frac{\tan ^7\theta _0}{y^7}, \quad \tan \theta _0+\delta<y\le 1, \\ \frac{1}{\sqrt{2}}-\frac{y-\tan \theta _0}{2\sqrt{2}\tan \theta _0} +\frac{(y-\tan \theta )^2}{8\sqrt{2}\tan ^2\theta _0}\\ +\frac{(y-\tan \theta )^3}{16\sqrt{2}\tan ^3\theta _0} - \frac{13 (y - \tan \theta _0)^4}{128 \sqrt{2}\tan \theta _0^4},\qquad \frac{\tan \theta _0}{2}<y\le \tan \theta _0+\delta , \\ 1-\frac{y^2}{2\tan ^2\theta _0}+\frac{3y^4}{8\tan ^4\theta _0}-\frac{5y^6}{16\tan ^6\theta _0},\qquad \epsilon <y\le \frac{\tan \theta _0}{2}. \end{array}\right. } \end{aligned}$$

By substituting Eq. (51) into Eq. (43), integrating and Taylor expanding the exponential, we find the leading-order terms of $L_C$ to be,

52 $$\begin{aligned} L_C(y)={\left\{ \begin{array}{ll} b_1\left( 1+\frac{\tan ^2\theta _0}{2\hat{y}^2}\right) , \hspace{12mm} \tan \theta _0+\delta<y\le 1, \\ \frac{b_2}{y^{b_0}} (1-H(y)+\frac{H(y)^2}{2}), \frac{\tan \theta _0}{2} \,\,<y\le \tan \theta _0+\delta , \\ \frac{b_3}{y}\left( 1+\frac{y^2}{2\tan ^2\theta _0}\right) , \quad \epsilon \,\le y\le \frac{\tan \theta _0}{2}, \end{array}\right. } \end{aligned}$$

where $b_0$, $b_1$, $b_2$ and $b_3$ are constants and *H*(*y*) is a function of the form $\sum _i p_i\left( \frac{y}{\tan \theta _0}\right) ^{q_i}$, with the $q_i$s being integers; all $b_i,p_i,q_i$ and *H* are defined in appendix F.

**Table 1:** Model parameter values

Parameter	Value	Description	References
$\nu _C^*$	140*Gpa*	Young’s modulus of cellulose	[45–48]
$\nu _H^*$	7*Gpa*	Young’s modulus of hemicellulose	[45–48]
$\theta _0$	0.1	Cellulose’s initial angle at deposition	[49]
$\breve{k}_0$	1	$\breve{k}_0=\frac{k_0^*}{k_{0,\text {hot}}^*}$, hotspots & hemicellulose breakage	
		rates ratio when unstressed	
$\varsigma _{\text {hot}}$ & $\varsigma$	1	$\varsigma _{\text {hot}}=\varsigma =\frac{ \kappa ^* L_0^{*2}}{2k_b^* T^*}$, a breakage rate constant	[35]
$\frac{n_{\text {hot},0}^* n_0^*}{\rho ^*}$	0.5	Ratio of fibre densities in $a_2$	[50, 51]
$\mu _0^*$	0.15	The initial viscosity	[52]
$\mu _1^*$	0.05	The target viscosity	[52]
$\epsilon$	0.0001	Cut-off value for the outer region of	
		the cell wall	
$a_1$	$0.1-0.6$	$a_1=A^*n_{\text {hot},0}^*$, cellulose stress modification	
		from hotspot crosslinking	
$a_2$	0.025	$a_2\frac{n_0^* n_{0,\text {hot}}^*\nu _H^*}{\rho ^* \nu _C^*}$, cellulose and hemicellulose stiffness	
		densities ratio	

In the model, we will be using the relevant parameters for the roots of *Arabidopsis thaliana*. We assume $\breve{k}_0=\frac{k_0^*}{k_{0,\text {hot}}^*}\approx 1$ so that the breakage rates of the hotspots and the hemicellulose fibres at rest are approximately the same. We set $a_1=0.1$ in most simulations as this number does not affect the results apart from the expansin action simulations where we shall comment on the affect of this parameter and its implications in Sect. 4

The stress resultant consists of three terms each representing a physical effect. Firstly, the cellulose contribution independent of the hotspots connections is:

53 $$\begin{aligned} \Sigma _1^\infty =\int ^1_\epsilon (L_c-1)\sin \theta \ \text {d}y= \sum _i c_{i}, \end{aligned}$$

where $c_i$ are constants dependent on $\tan \theta _0$ or $\epsilon$ (defined in Table 2). Secondly, the cellulose contribution when crosslinked by the hotspots is:

54 $$\begin{aligned} \Sigma _2^\infty&= \int ^1_\epsilon a_1 n_{\text {hot}} (L_c-1)\sin \theta \ \text {d}y\nonumber \\&=a_1\left( \left( \sum _i d_{1,i}(\alpha )\right) + \alpha \tan _0\left( b_1-1\right) \right. \nonumber \\&+ \left( \sum _i d_{2,i}(\alpha )\right) (\delta + \tan \theta _0)^{\frac{1}{\alpha }}\nonumber \\&+ \alpha \tan \theta _0\left( 1-b_1\right) (\delta + \tan \theta _0)^{\frac{1}{\alpha }}\nonumber \\&+ \left( \sum _i d_{3,i}(\alpha )\right) \left( \frac{\tan \theta _0}{2}\right) ^{\frac{1}{\alpha }}+ b_3 \alpha \left( \frac{\tan \theta _0}{2}\right) ^{\frac{1}{\alpha }}\nonumber \\&\left. +\left( \sum _i d_{4,i}(\alpha )\right) (\chi _{\text {hot}})^{\frac{1}{\alpha }} -\alpha b_3 (\chi _{\text {hot}})^{\frac{1}{\alpha }}\right) . \end{aligned}$$

The generic form of the constants $d_{j,i}$ is $\frac{w_1}{w_2\left( \frac{1}{\alpha }+w_3\right) }$, where $w_1, \ w_2$ and $w_3$ are known constants often dependant on $\tan \theta _0$, $\delta$ and $\chi _{\text {hot}}$ (see Table 3). Lastly, the contribution from the hotspots is then

55 $$\begin{aligned} \Sigma _3^\infty =&\int ^1_\epsilon a_2 n n_{\text {hot}} \sigma _H \ \text {d}y \nonumber \\&=a_2\breve{\alpha }\left( \frac{\alpha }{\breve{k}_0+1}\left( 1-\chi ^{\frac{\breve{k}_0+1}{\alpha }}\right) \right. \end{aligned}$$

56 $$\begin{aligned}&\left. -\frac{1}{\frac{1}{\alpha }\left( \breve{k}_0+\omega +1\right) +1} \left( 1-\chi ^{\frac{\breve{k}_0+\omega +1}{\alpha }+1}\right) \right) . \end{aligned}$$

Further simplifications to the stress resultant can be made by examining the cases for small and large strain rate, $\alpha \ll 1$ and $\alpha \gg 1$, respectively. When $\alpha \ll 1$, all terms of the form $\mathcal {Z}^{\frac{1}{\alpha }}$ that appear in the expansions are negligible, since $\mathcal {Z}$ is less than one for all such expressions. When $\alpha \gg 1$, all the terms of the form $\frac{w_1}{w_2\left( \frac{1}{\alpha }+w_3\right) } \mathcal {Z}^{\frac{1}{\alpha }}$ are negligible compared to terms of the form $\alpha \mathcal {Z}^{\frac{1}{\alpha }}$. As a result,

**Fig. 3:**
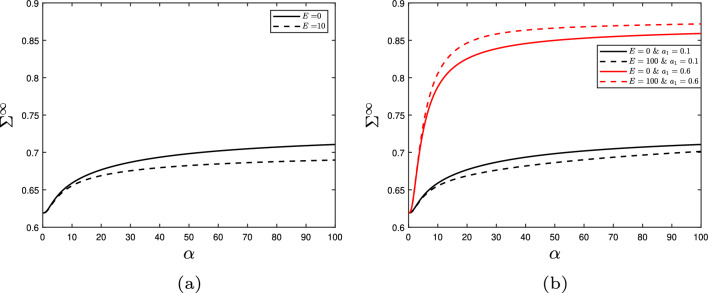
The effect on the stress resultant $\Sigma ^\infty$ plotted against the strain rate $\alpha$ of expansin acting with rate *E* on **a** the resting length, $L_0$, which decreases the stress resultant and thus the yield threshold; or **b** the viscosity, $\mu$, where the small (large) $a_1$ value represents reduced (enhanced) cellulose crosslinking by the hotspots and hence decreased (increased) the stress resultant and yield threshold. Other parameters: $\beta _{\text {hot}}=0.01$, $\gamma =0.05$, and others found in Table 1

57 $$\begin{aligned} \Sigma ^\infty =\left\{ \begin{array}{lllll} \sum _i c_{i} + a_1\alpha \tan \theta _0\left( b_1-1\right) \\ \hspace{3mm}+a_2\breve{\alpha }\left( \frac{\alpha }{\breve{k}_0+1} -\frac{1}{\frac{1}{\alpha }(\breve{k}_0+\omega +1)+1}\right) , &  \quad \alpha \ll 1,\\ \sum _i c_{i} + a_1\alpha \left( \tan \theta _0\left( b_1-1\right) \right. \\ \left. \hspace{3mm} + \tan \theta _0\left( 1-b_1\right) (\delta + \tan \theta _0)^{\frac{1}{\alpha }} \right. \\ \left. \hspace{3mm} + b_3 \left( \frac{\tan \theta _0}{2}\right) ^{\frac{1}{\alpha }} -b_3 (\chi _{\text {hot}})^{\frac{1}{\alpha }}\right) \\ \hspace{3mm} +a_2\breve{\alpha }\left( \frac{\alpha }{\breve{k}_0+1}\left( 1-\chi ^{\frac{\breve{k}_0+1}{\alpha }}\right) -\frac{1}{\frac{1}{\alpha }\left( \breve{k}_0+\omega +1\right) +1}\right) , \hspace{5mm}\\ & \quad \alpha \gg 1. \end{array} \right. \end{aligned}$$

Equation (57) gives the leading-order terms that control the dominant cell wall behaviour for small and large strain rates, neglecting the effect of expansin action. This expression is used to validate the numerical scheme presented in Sect. 4, where we also discuss the biological implications.

## Results and discussion

The complexity of the plant cell wall and its growth process present many modelling challenges. We have therefore created a simple model to focus our attention on the cell wall structure and its implications on possible enzyme action.

The system of Eqs. (38)–(44) is solved for the bond number and densities *n* and $n_{\text {hot}}$, the CMF angle $\theta$, and the stress resultant $\Sigma$, subject to a choice of expansin action where $\sigma _H$ is determined by one of the Eqs. (34)–(37). Where the steady-state stress resultant is required, we use (45) instead of (44). The asymptotically simplified expressions (53)–(57) for the steady-state stress resultant are used where applicable. This study focusses on the cellulose crosslinking dynamics; hence, we neglect the effect of pectin on the cell wall dynamics by setting $\Gamma =0$ throughout. All other fixed parameter values are listed in Table 1. All solutions are obtained using MATLAB; in particular, the integrals are computed numerically using the Legendre–Gauss Quadrature code by Greg von Winckel [44].

We begin the discussion of the results by first analysing the model’s implications on the cell wall structure and yield threshold in Sect. 4.1. Secondly, we explore possible enzyme mechanisms and the likelihood of hotspots structures in the cell wall in Sect. 4.2. We finish with some final remarks in Sect. 4.3.

### Implications for cell wall structure

Before delving into the results, we outline the logic as to how the stress resultant links to the cell wall yield threshold and growth rate using expansin’s effect as an example. A fixed turgor pressure in a growing cell wall (constant strain rate $\alpha$) will result in a specific cell wall tension and therefore a set stress resultant. When expansin acts on the hotspots resting length, $L_0$, the same cell wall stress/turgor pressure produces different strain rates (growth rate) (Fig. 3a, solid line versus dashed line); Eq. (1) then implies that the yield threshold has changed. We know this from the plateauing effect; the extensibility determines the gradient, but it is the yield threshold that scales the plateau up and down as $\alpha \gg 1$. Further analysis of the stress–strain-rate relationship and the crosslinks influence on the cell-wall yield threshold can be found in [35]. For the purpose of this article, it suffices to know that a decrease in stress resultant implies a decrease in the yield threshold.

From the collective results presented here, we observe some general trends. As the strain rate increases, the stress resultant plateaus (e.g. Fig. 3). This relationship arises due to the bond number and densities (*n*, $n_{\text {hot}}$) behaviour when $\alpha$ is changed, despite the inclusion of linear elasticity. Specifically, for low values of $\alpha$, the bond density $n_{\text {hot}}$ remains low for much of the domain (see Fig. 6b). However, as $\alpha$ is increased, the bonds become increasingly loaded and remain intact for longer (increasing the overall stress held in the cell wall) before finally breaking. Eventually the hotspots reach a limit as to how much stress they can withstand without breaking, and the bond density drops to 0 when $y\approx 0$; the amount of stress held across the cell wall has reached capacity (Fig. 6b). Reaching this capacity consequently limits further increases to the stress resultant, implying that the cell wall’s yield threshold has a maximum, where further increasing the growth rate has limited effect. This result demonstrates that cell wall strength is capped by the mechanism of cellulose crosslinkage, despite the cellulose fibres being the major load-bearing component in the cell wall.

**Fig. 4:**
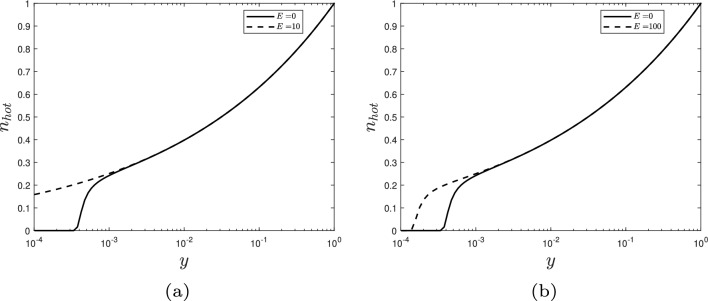
The effect on the hotspots density, $n_{\text {hot}}$ through the cell wall thickness *y* of expansin with rate *E* acting on **a** the resting length, $L_0$; or **b** the viscosity, $\mu$. Parameters: $\alpha =5$, $\beta _{\text {hot}}=0.01$, $\gamma =0.05$, and others found in Table 1. In both cases, expansin has increased the bond densities and thus has not weakened the cell wall

We now examine the effect on the stress resultant of cellulose reorientation during growth. For lower strain rates, we find that the stress resultant increases as time progresses (Fig. 7a) due to a combination of all the fibres being increasingly strained and the cellulose reorientating. For larger strain rates, $\alpha$, the stress has very little variation over time because the large reorientation rate allows fibres to converge to the same distribution. For smaller times, the stress has a sharp maximum (Fig. 7a). This peak occurs because increasing the strain rate stretches and reorients the fibres uniformly, due to the equivalent effect of $\alpha$ and *t* on the fibres from the term $e^{\alpha t}$. On the other hand, fibres deposited at $t > 0$ are progressively stretched and rearranged as they move to the outside of the wall. Thus, for certain times/strain rates (between $\alpha =10-30$), the sum of the stress arising in this family of fibres is less than those present at $t = 0$. This raised stress resultant does not last as the crosslinks present at $t=0$ eventually break, causing a rapid decrease in the cell wall strength and producing a sharp peak in the stress (Fig. 7a). The effect of cellulose reorientation can also be seen when the initial fibre angle, $\theta _0$, is increased (Fig. 7b). According to Eq. (42), for any fixed fibre position *y*, the CMF increasingly reorient as $\theta _0$ increases. Consequently, increasing $\theta _0$ also significantly increases the stress resultant (Fig. 7b). From these results, we conclude that cellulose reorientation leads to a higher stress resultant in the plant cell wall and, subsequently, increases the cell wall yield threshold. The yield threshold increasing due to cellulose reorientation could provide a possible mechanism for growth slow-down and hence the beginning of the cell’s secondary wall structure. This hypothesis has been previously put forward [19]. Cellulose reorientation reducing axial growth has been previously reported in other mathematical models [53, 54].

We now proceed to analyse the implications of the asymptotic reduction of the stress resultant. There is substantial agreement between the asymptotic expansions and the numerical results, for all three combinations of $\beta _{\text {hot}}$ and $\gamma$ (Fig. 8). There is little difference in accuracy between the full asymptotic Eqs. (53)–(56) and the simplified expansion (57), demonstrating that the cell wall yield threshold’s behaviour is dominated by the terms in the simplified equation. According to Eqs. (53) and (54), the constants $c_i$ and $d_{j,i}$ in the cellulose’s contribution to the cell wall strength depend on $\tan \theta _0$ (Table 2), whereby increasing $\tan \theta _0$ increases the stress held in the cell wall (Fig. 7). Since $a_2<a_1$ (see Table 1), we conclude that the main control of the cell wall yield threshold in the axial direction is the cellulose orientation. This result matches past simulation results [17] and the current consensus that the cellulose orientation controls the growth direction in most plant cells [21].

In Eq. (57) that characterises the cell wall stress behaviour, the hotspots density’s contribution (the $\chi _{\text {hot}}$ term) is controlled by $b_3$. This coefficient determines the slope of the extension curve of the cellulose molecules, $L_C$, as they rapidly extend close to $y=0$ where they are most stressed. Thus, the effect on the yield threshold of breaking the hotspot crosslinks (changing $\beta _{\text {hot}}$) is predominantly actioned through loosening the smaller group of significantly stressed cellulose molecules.

Equation (57) also demonstrates why the stress resultant plateaus (Fig. 3). Rearranging the equation gives

58 $$\begin{aligned} \Sigma ^\infty =&\sum _i c_{i} \nonumber \\&+a_1\alpha \left( \tan \theta _0\left( b_1-1\right) \left( 1- (\delta + \tan \theta _0)^{\frac{1}{\alpha }}\right) \right. \nonumber \\&\left. + b_3 \left( \left( \frac{\tan \theta _0}{2}\right) ^{\frac{1}{\alpha }} - (\chi _{\text {hot}})^{\frac{1}{\alpha }}\right) \right) \nonumber \\&+a_2\breve{\alpha }\left( \frac{\alpha }{\breve{k}_0+1}\left( 1-\chi ^{\frac{\breve{k}_0+1}{\alpha }}\right) \right. \nonumber \\&\left. -\frac{1}{\frac{1}{\alpha }\left( \breve{k}_0+\omega +1\right) +1}\right) . \end{aligned}$$

As $\alpha \rightarrow \infty$, the first term on the first line remains constant. The second term can be recognised and reexpressed to be a limit of the form $\lim _{x\rightarrow 0} \left( \frac{f^x-1}{x}\right) \rightarrow \ln f$ and therefore is a constant. The last term also converges to a constant (with $\breve{\alpha }$ also converging to a constant) for the same reason. Collectively this means that as $\alpha$ increase the stress resultant will level out and become constant. We also note that for $\alpha \ll 1$, the stress resultant is not controlled by $\chi$ and $\chi _{\text {hot}}$ at all. This implies for low levels of turgor pressure, enzyme action on the cell wall is ineffective at controlling the growth rate and that it is solely controlled by the orientation and concentration of fibres.

### Enzyme action on the cell wall

We first discuss how our simulations can be directly compared to experimental results relating to enzyme action. Experiments have shown upon overexpressing or silencing expansins, the growth (through elongation and division) of plant roots and leaves increase or decrease respectively in a variety of species [55, 56], and root hair and leaf primordia initiation are also affected [57]. Overexpression and knockout mutations were also performed on XTH proteins which made little difference to growth [58, 59]. Moreover, inactivating and then extending plant tissues while applying Cel12A or expansins permitted cell wall extension [10, 28]. The effect on growth and hence the strain rate of enzyme action can be seen in our model through the change in stress resultant. A decrease in the stress resultant will lead to an increased growth rate (strain rate) and vice versa as described in Sect. 4.1. Additionally, from the extension experiments with multiple deformations (elastic and plastic) and examining the stress relaxation response, expansin was shown not to weaken the wall while Cel12A did. Additionally, XTH was found to have only minor effects on cell wall mechanics [58]. This effect can be directly observed in the model by examining the bond density distributions; if there are fewer bonds intact, the wall integrity will be weaker.

We now examine different modes of enzyme action beginning with expansin. As previously mentioned in Sect. 1, expansin causes wall loosening without reducing the strength of the cell wall, potentially by allowing the slippage of fibres within the hotspot. We simulated expansin’s hypothesised action via two methods: increasing the spring resting length and decreasing the viscosity of the dashpot by equations characterised in Sect. 2.2.3 (Eqs. 35 and 37). Simulations of expansin’s theorised fibre slippage action on the resting length (Eq. 35) reveal a decrease in the stress resultant (Fig. 3a), while ensuring the bond density does not reach zero (Fig. 4a). Similarly, expansin’s slippage action on the viscosity of the dashpot (Eq. 37) decreases the stress resultant provided hotspots cellulose crosslinking is small (Fig. 3b) and always increases the bond density (Fig. 4b). This bond density increase arises due to stress relaxation; both mechanisms reduce the elastic strain imposed on the fibres which are therefore less likely to break. Two opposing effects contribute to the overall stress resultant: stress relaxation from the hotspots, combined with the inclusion of more, increasingly stretched, CMF molecules towards the outer boundary of the cell wall. When the hotspots-cellulose crosslinking is sufficiently large to counteract the hotspots stress relaxation, the stress resultant increases with expansin action due to the increased contribution of the CMF (Fig. 3b). Therefore, both mechanisms can decrease the axial stress resultant for all strain rates (Fig. 3) and are consequently effective at inducing growth. Moreover, the hotspots bond densities $n_{\text {hot}}$ increase for both enzyme action pathways (Fig. 4), increasing cell wall integrity and thus strengthening the cell wall. So, allowing the fibres to slide past one another (the dashpots) or relax (increasing resting lengths) loosens the fibre network and decreases the likelihood of the bonds breaking. This explains the observed experimental behaviour (as described in Sect. 1) and provides evidence for expansin’s hypothesised slippage action.

**Fig. 5:**
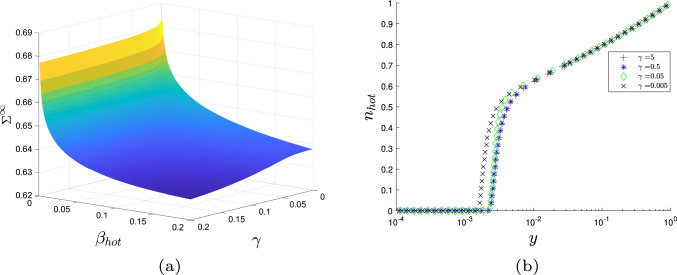
Enzyme cutting action. **a** The effect on the stress resultant, $\Sigma ^\infty$ of Cel12a and XTH with $\alpha =50$. Cel12a’s action, $\beta _{\text {hot}}$, on the whole hotspot is much more effective at decreasing the stress resultant than XTH’s action, $\gamma$, on the hemicelluloses. **b** XTH’s action on the hotspots density $n_{\text {hot}}$ with $\beta =0.1$ and $\alpha =10$ showing its limited impact on the hotspot density. All other parameters can be found in Table 1

**Fig. 6:**
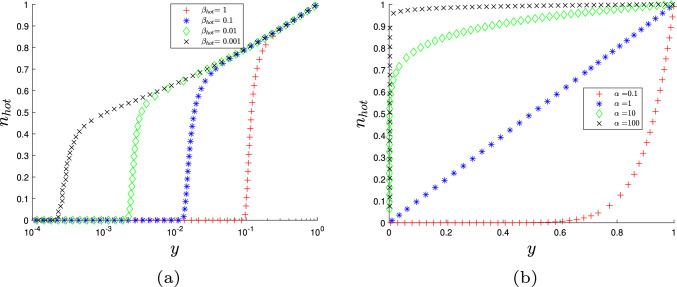
The hotspot density, $n_{\text {hot}}$, parameter dependence. **a** The effect of varying Cel12a’s action on $n_{\text {hot}}$ with $\alpha =10$ demonstrating its effectiveness at decreasing the hotspot density. **b** The effect of varying $\alpha$ with $\beta _{\text {hot}}=0.01$ on $n_{\text {hot}}$, both with $\gamma =0.05$, showing the transitional behaviour of increasing $\alpha$ and the eventual plateauing under further increase. All other parameters can be found in Table 1

**Fig. 7:**
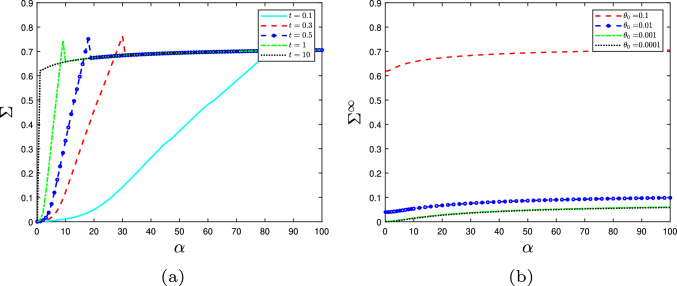
Changes in the stress resultant as we effect the cellulose fibre orientation by changing **a** the time point and **b** the cellulose angle at deposition, $\theta _0$ with $\beta =0.01$ and $\gamma =0.05$. Both graphs demonstrate that as the cellulose fibres increasingly reorient, they increase the stiffness and yield threshold in the axial direction. All other parameters can be found in Table 1

In contrast, when the parameter $a_1$ is increased the hypothesised expansin action causes the yield threshold to increase (Fig. 3b) despite the loosening of the fibre network. This observation may be consistent with biological observations. It has been observed that plants lacking xyloglucan have only a small amount of growth reduction and alteration in cell wall structure [14, 60]. It has therefore been suggested that any xyloglucan present is concentrated in these compact biomechanical hotspots. Therefore, as $a_1$ represents the proportion of xyloglucan in the wall, we find that in order to recreate expansin’s experimental behaviour, there must be a small amount (comparatively to the cellulose) of xyloglucan that is concentrated around the hotspots.

Continuing the investigation of hypothesised enzyme action, we now focus on XTH and Cel12A. As detailed in Sect. 1, XTH and Cel12A are theorised to hydrolyse hemicellulose and the hotspots respectively. Recall that we recreate this cutting action by increasing the breakage rate $\gamma$ of the hemicellulose fibres to model XTH, and increasing the hotspot breakage rate $\beta _{\text {hot}}$ to model Cel12A action. By testing a range of values for both parameters, we discover that $\beta _{\text {hot}}$ significantly affects the stress resultant compared to $\gamma$ (Fig. 5a), (Cel12A’s cellulose digestion effect is investigated in appendix E where it simply increases its effectiveness on the stress resultant). These results agree with previous work, which also finds the yield threshold to be controlled by the rate of dissociation of tethering crosslinks [61]. The effect of $\gamma$ on the hotspot bond density, $n_{\text {hot}}$, is also negligible (Fig. 5b). In contrast, an increased $\beta _{\text {hot}}$ leads to a considerable decrease in $n_{\text {hot}}$ (Fig. 6a). As the fibres move towards the outer $y=0$ boundary, their lengths increase sharply ($L=\frac{1}{y}$), leading to significantly larger stress; the stress resultant is therefore highly sensitive to small changes in the transition location $\chi _{\text {hot}}$ in the region $0<y<0.1$ where $n_{\text {hot}}$ drops rapidly. Thus, increasing the breakage rate of the hemicellulose fibres (increasing $\gamma$) is ineffective when compared to targeting the hotspots themselves (increasing $\beta _{\text {hot}}$).

Simulated XTH action generates minimal reductions in the stress resultant and the hotspots density, implying that minimal growth is induced due to its limited wall-loosening ability. This effect occurs despite a natural assumption that the hydrolysis of bonds by some members of the XTH family could cause wall loosening [10, 28]. The model outputs offer two interpretations to explain this phenomenon: either the hotspots maintain their integrity when hemicellulose fibres are cut, or hydrolysing hemicellulose could be ineffective at breaking hotspots down if the hotspots are compact and inaccessible. Thus, the limited wall loosening is a consequence of the cell wall retaining its integrity, with minimal increase in cell wall growth as observed in experiments [10]. In the asymptotic expansions (Eqs. (57)), it is $\beta _{\text {hot}}$, not $\gamma$ (in the form of $\chi _{\text {hot}}$), that controls the leading-order terms of cellulose’s contribution. We therefore explicitly see that if XTH’s ability to break down the hotspots is ineffective, the observed experimental behaviour is reproduced.

By recreating Cel12A’s hypothesised hotspot-cleaving action, we see that it weakens the cell walls by decreasing the hotspots density, which decreases the stress resultant and induces growth, matching biological experiments [10]. This effect would not occur if a tethered network was assumed, in which case the model would only have one bond density that is not dependent on another (in a similar manner to [35]), leaving us unable to distinguish between Cell12A and XTH’s action. Through analysis of the breakage rates and the model’s assumption of the hotspots hypothesis, we have recreated experimentally observed enzyme behaviour.

**Fig. 8:**
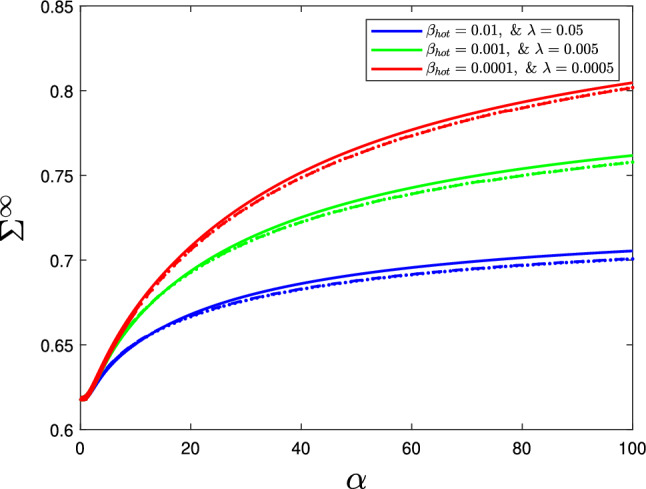
Comparison between the axial stress resultant’s numerical solution (solid line) and asymptotic approximations, Eqs. (53)–(56) (dashed line) and (57) (dotted line), at various values of $\beta _{\text {hot}}$ and $\gamma$, demonstrating the accuracy of the asymptotic expansions. All other parameters can be found in Table 1

### Remarks

The results presented play into the hypothesis [27] first stated that growing plant cell walls seem to undergo a glass transition. This theory suggests that hemicellulose connecting the cellulose molecules is not the primary stress-bearing component (as $a_1$ is small) and holds the wall in tension close to the yield threshold, such that when the stress relaxation imposed on the hotspots, by digestion or slippage occurring, it could be enough to cause the redistribution of stress to other cell wall components such as pectin (and its linkers with cellulose) and hence induce growth. Thus, we have a rapid change in viscosity with the extra load causing the pectin to ‘melt’ and flow. As the cell wall extends, other hotspots could become strained, hence raising the yield threshold and requiring further relaxation to continue growth.

The results in this article depend on the inclusion of the cellulose contributing to the axial stress. Even though, on average, the cellulose molecules are perpendicularly oriented to the growth axis [22], the findings show the importance of including cellulose reorientation or a distribution of angles in mathematical models of growth.

There is still further modelling work to be done. We have only examined axial growth, so it remains to be understood how enzyme action does not necessarily lead to radial growth and the consequences of cellulose reorientation, as it has been observed a reduction in anisotropy can lead to radial swelling [62]. Expansin has been shown to induce growth as soon as it is applied, while Cel12A-induced growth is delayed after application [12]. Our model cannot explain this time delay phenomenon, meaning we could benefit from further work on the crosslinking dynamics to understand this process. Many experiments are done on a tissue level, while our model examines only a small cell wall section. Therefore, there could be advantages to constructing a tissue-scale model for cells, including their interactions and the different cell layers to match with experiments. Models such as [17] also have great potential to aid our understanding but currently neglect growth and enzyme manipulation. The stress–strain relationship from this model could be used to inform constitutive laws and then incorporated into a larger-scale model with growth and wall modifications.

**Fig. 9:**
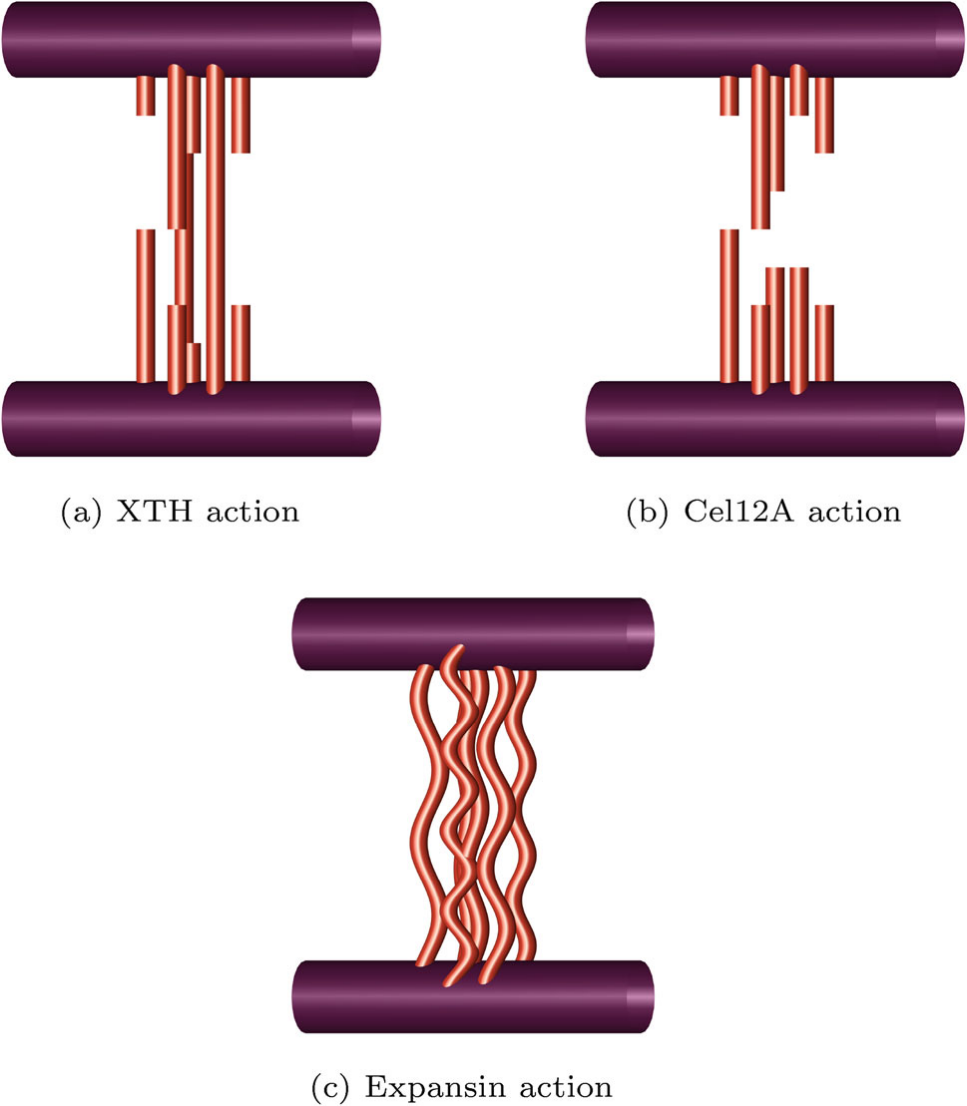
Hypothesised enzyme action. The purple rods represent cellulose molecules, and the red rods are the hemicellulose fibres in a hotspot. **a** XTH hydrolysis action only cutting a select amount of hemicellulose fibres. **b**) Cel12A hydrolysis action cutting the whole hotspot. **c** Expansin loosening action allowing the hotspot to be intact while allowing extension

## Conclusion

By deriving and analysing a mathematical model of the cell wall incorporating the biomechanical hotspot dynamics as proposed by [9], we have explained experimentally observed enzyme behaviour and thus provided insight into the cell wall structure and loosening mechanisms. We propose the following enzyme mechanisms in line with experimental observations (Fig. 9). XTH only cuts individual hemicellulose fibres and thus does not provide sufficient wall loosening to induce growth, possibly due to the hotspots remaining inaccessible or hemicellulose scissoring being insufficient to fully break down the hotspot. On the other hand, Cel12A can cleave the whole hotspot allowing the cell to grow while also weakening the cell wall. Expansin action causes fibre slippage, permitting stress relaxation, enabling cell wall extension without weakening the cell wall. We have therefore presented a mechanistic understanding of potential enzyme action. Finally, cellulose reorientation during cell wall extension leads to a decrease in the axial growth rate and its range orientation is the primary control for cell growth and the yield threshold.

Enzyme action on the cell wall is an under-researched area of plant growth often overlooked by modellers. The theory presented in this paper generates improved understanding of the fundamental mechanisms underlying plant cell growth. We hope that it provides a key building block towards a unified framework for plant development.

## Figures and Tables

**Fig. 10:**
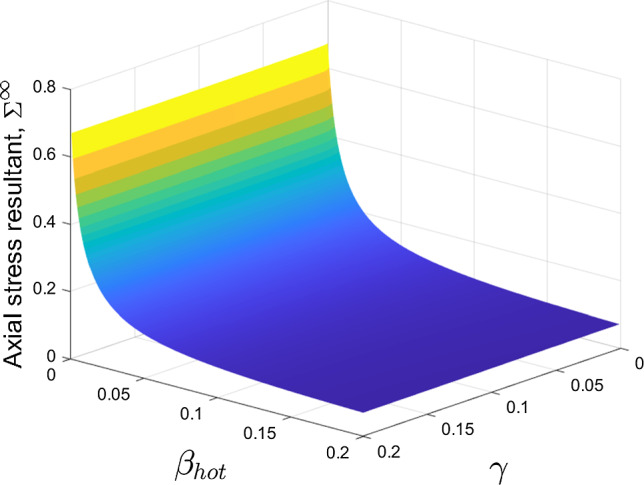
Enzyme cutting action. The effect on the stress resultant of Cel12a with its additional cellulose digestion effect and XTH with $\alpha =50$, $\tilde{C}=100$ and $\mathcal {N}=10$. Cel12a’s action, $\beta _{\text {hot}}$ and its degradation, on the whole hotspot is much more effective at decreasing the stress resultant than XTH’s action, $\gamma$, on the hemicelluloses. All other parameters can be found in Table 1

## Data Availability

There were no data used in the paper, therefore none is available.

## Appendix A: Proving constant density

The density at any point, $\rho ^*(x^*,y^*,t^*)$, is described by

A1 $$\begin{aligned} \frac{\partial \rho ^*}{\partial t^*}+ \textbf{u}^*\cdot \nabla ^* \rho ^* =0, \ \text {with} \ \rho ^*(x^*,h^*,t^*)=\rho _0^*\qquad \end{aligned}$$

where $\rho _0^*$ is the density at which material is deposited. Using the total derivative, we have $\frac{d \rho ^*}{d T^*}=0$ on the characteristics $\frac{d x^*}{d t^*}=\alpha ^* x^*$ and $\frac{d y^*}{d t^*}=-\alpha ^* y^*$ from Eq. (2). With using the initial conditions $(x^*,y^*)=(x_0^*,h^*)$ at $t^*=\tau ^*$ for some height up the cell, $x_0^*$ and some time $\tau ^*\ge 0$ we obtain $x^*=x_0^* e^{\alpha ^*(t^*-\tau ^*)}$ and $y^*=h^*e^{-\alpha ^*(t^*-\tau ^*)}$ for $t^*>\tau ^*$. Therefore along the streamline $x^*y^*=x_0^*h^*$, we have $\rho ^*=\rho _0^*$. Consequently if $\rho ^*(x^*,y^*,0)=\rho _0$ for $0<y^*<h^*$, the density remains uniform for $t^*>0$ meaning we can assume the density of the cell wall components remains uniform for all time as well.

## Appendix B: Solving stress terms

We first solve Eq. (25) to find the axial hemicellulose stress when not acted upon by any expansin action ($L_0$ and $\mu$ are constant). By using Eq. (24), we have $\frac{\partial s}{\partial t}=\frac{\text {d}L}{\text {d}t}=\alpha L$, and as a result, we can rewrite Eq. (25) as,

B2 $$\begin{aligned} \frac{\partial \sigma _{H}}{\partial t}+\omega \sigma _{H}=\alpha L(t). \end{aligned}$$

If we shift the time parameter so that $\bar{t}=t-\tau$ where the fibre is deposited at time $t=\tau$, we have that $\sigma _H(1,0)=0$. The above then becomes,

B3 $$\begin{aligned} \frac{\partial \sigma _{H}}{\partial \bar{t}}+\omega \sigma _{H}=\alpha L(\bar{t}). \end{aligned}$$

Using an integrating factor and integrating both sides, we derive,

B4 $$\begin{aligned} \sigma _{H}=e^{-\omega \bar{t}}\int ^{\bar{t}}_0 \alpha L(\hat{t})e^{\omega \hat{t}} \ \text {d}\hat{t}, \end{aligned}$$

where on substituting $L=e^{\alpha \bar{t}}$ (using boundary conditions $L(0)=1$) we then have,

B5 $$\begin{aligned} \sigma _{H}=\frac{\alpha }{\alpha +\omega }e^{-\omega \bar{t}}\left( e^{(\alpha +\omega )\bar{t}}-1\right) . \end{aligned}$$

The fibres follow the trajectories $y=e^{-\alpha \bar{t}}$ (from Sect. 2 using boundary condition $y(0)=1$) and with $y=\left( e^{\omega \bar{t}}\right) ^{-\frac{\alpha }{\omega }}=\frac{1}{L}$, we calculate the final form as

B6 $$\begin{aligned} \sigma _H=\frac{\alpha }{\alpha +\omega }\left( L-L^{-\frac{\omega }{\alpha }}\right) . \end{aligned}$$

For the expansin action on the spring resting length (Eq. (32)), we were unable to find an analytic expression, as a result we use forward finite differences to solve the equation. We need this expression in terms of *y* so we first divide it by $\frac{\text {d}y}{\text {d}\bar{t}}=-\alpha y$ to get

B7 $$\begin{aligned} \frac{\text {d}L_0}{\text {d}y}=\frac{E}{-\alpha y}\left( \frac{1}{yL_0}-1\right) . \end{aligned}$$

We partition the domain such that $y=\{y_1,y_2,\ldots y_m\}$ where $y_1=0$ and $y_m=1$ and $L_0(y_i)=L_{0,i}$ for $i=1\ldots m$ to get the recursive relation

B8 $$\begin{aligned} L_{0,i}&=L_{0,i+1}+\frac{E}{\alpha y_{i+1}}(y_{i+1}-y_i)\left( \frac{1}{y_{i+1}L_{0,i+1}}-1\right) \ \nonumber \\&\quad \text {where} \ L_{0,m}=1. \end{aligned}$$

We can then use the calculated values of $L_0$ in Eq. (32) to find the derivative of $L_0$. We now rederive the hotspot stress, $\sigma _H$ with non-constant resting length using the initial equation (Eq. 25), shifting time again and the fact that $\frac{ds}{\text {d}\bar{t}}=\frac{1}{L_0}\frac{\text {d}L}{\text {d}\bar{t}}-\frac{L}{L_0^2}\frac{\text {d}L_0}{\text {d}\bar{t}}$, to get,

B9 $$\begin{aligned} \frac{\partial \sigma _{H}}{\partial \bar{t}}+\omega \sigma _{H}=\frac{\alpha L}{L_0}-\frac{L}{L_0^2}\frac{\partial L_0}{\partial \bar{t}}, \end{aligned}$$

and using the same method as above and Eq. B8 we calculate,

B10 $$\begin{aligned} \sigma _H=L^{-\frac{\omega }{\alpha }}\int ^{\bar{t}}_0 L^{\frac{\omega }{\alpha }} \left( \frac{\alpha L}{L_0(L)}-\frac{L}{L_0^2(L)}\frac{\text {d}L_0(L)}{\text {d}\hat{t}}\right) \ \text {d}\hat{t}. \end{aligned}$$

which is the hemicellulose stress term for enzyme action on the resting length, $L_0$.

We repeat the same procedure for expansin action on the impedance, $\mu$. By solving Eq. (31), we can obtain,

B11 $$\begin{aligned} \mu =\frac{1}{\mathcal {M}+(1-\mathcal {M})e^{-Et}}. \end{aligned}$$

This means that $\omega =\frac{\nu _H^*}{\mu _0^*\mu k_{0,\text {hot}}^*}=\omega _0\left( \mathcal {M}+(1-\mathcal {M})e^{-Et}\right)$ where $\omega _0=\frac{\nu _H^*}{\mu _0^*k_{0,\text {hot}}^*}$. Substituting this into the non-dimensional stress term (Eq. (25)) and shifting time we have,

B12 $$\begin{aligned} \frac{\partial \sigma _{H}}{\partial \bar{t}}+\omega _0\left( \mathcal {M}+(1-\mathcal {M})e^{-E\bar{t}}\right) \sigma _{H}=\alpha L, \end{aligned}$$

where we again use an integrating factor and $L=e^{\alpha \bar{t}}$ to find

B13 $$\begin{aligned}&\exp \left( \omega _0\left( \mathcal {M} \bar{t}-\frac{1}{E}(1-\mathcal {M})e^{-E\bar{t}}\right) \right) \frac{\partial \sigma }{\partial \bar{t}}\nonumber \\&\qquad +\left( \omega _0\left( \mathcal {M}+(1-\mathcal {M})e^{-E\bar{t}}\right) \right) \nonumber \\&\qquad \times \exp \left( \omega _0\left( \mathcal {M} \bar{t}-\frac{1}{E}(1-\mathcal {M})e^{-E\bar{t}}\right) \right) \sigma \nonumber \\&\quad =\alpha e^{\alpha \bar{t}}\exp \left( \omega _0\left( \mathcal {M}\bar{t}-\frac{1}{E}(1-\mathcal {M})e^{-E\bar{t}}\right) \right) . \end{aligned}$$

Upon integrating we then get

B14 $$\begin{aligned} \sigma _H=&\exp \left( -\omega _0\left( \mathcal {M}\bar{t}-\frac{1}{E}(1-\mathcal {M})e^{-Et}\right) \right) \nonumber \\&\quad \times \int ^{\bar{t}}_0 \alpha \exp \left( \alpha \hat{t}\right) \exp \left( \omega _0\left( \mathcal {M}\hat{t}-\frac{1}{E}(1-\mathcal {M})e^{-E\hat{t}}\right) \right) \nonumber \\&\quad \times \ \text {d}\hat{t}, \end{aligned}$$

where we then make the substitution $\hat{y}=e^{-\alpha \hat{t}}$ in the integral. This implies that $e^{-Et}=y^{\frac{E}{\alpha }}$ and $\text {d}t=-\frac{1}{\alpha y} \text {d}y$ meaning

B15 $$\begin{aligned} \sigma _H&=y^{\frac{\omega _0\mathcal {M}}{\alpha }}\exp \left( \frac{\omega _0}{E}(1-\mathcal {M})y^{\frac{E}{\alpha }}\right) \quad \int ^1_y \alpha \hat{y}^{-2-\frac{\omega _0\mathcal {M}}{\alpha }}\nonumber \\&\times \exp \left( -\frac{\omega _0}{E}(1-\mathcal {M})\hat{y}^{\frac{E}{\alpha }}\right) \ \text {d}\hat{y}, \end{aligned}$$

## Appendix C: Solving the evolving bond densities

To determine the evolving bond number and densities we solve (30) for *n*, using the method of characteristics with $\frac{\text {d}y}{\text {d}t}=-\alpha y$, $\frac{\text {d}x}{\text {d}t}=\alpha x$, and $\frac{\partial n}{\partial x}=0$ meaning the left-hand side of Eq. (30) can be rewritten as,

C16 $$\begin{aligned}&\frac{\partial n}{\partial t} -\alpha y \frac{\partial n}{\partial y}+\alpha x \frac{\partial n}{\partial x}= \end{aligned}$$

C17 $$\begin{aligned}&\frac{\partial n}{\partial t} +\frac{\partial y}{\partial t}\frac{\partial n}{\partial y}+\frac{\partial x}{\partial t} \frac{\partial n}{\partial x}= \frac{d n}{d t}, \end{aligned}$$

the total derivative. As a result, Eq. (30) becomes

C18 $$\begin{aligned} \frac{dn}{\text {d}t}=-\breve{k}_0 n\exp (\gamma ^2\varsigma s_{H,e}^2). \end{aligned}$$

Combining Eqs. (24) and (C18), we derive

C19 $$\begin{aligned} \frac{dn}{\text {d}L}=-\breve{k}_0\frac{n}{\alpha L}\exp (\gamma ^2 \varsigma s_{H,e}^2), \end{aligned}$$

and integrating we obtain

C20 $$\begin{aligned} n=\exp \left( -\frac{\breve{k}_0}{\alpha }\int ^{L}_1 \frac{\exp (\gamma ^2 \varsigma s_{H,e}^2)}{\hat{L}} \ \text {d}\hat{L} \right) , \end{aligned}$$

which we can write as

C21 $$\begin{aligned}&n=\exp \left( -\frac{\breve{k}_0}{\alpha }G\left( \frac{1}{L}\right) \right) \ \text {with} \nonumber \\&G(y)=\int ^{1}_y \frac{\exp (\gamma ^2 \varsigma s_{H,e}^2)}{\hat{y}} \ \text {d}\hat{y}. \end{aligned}$$

To find the hotspot bond density, $n_{\text {hot}}$ (Eq. (29)), we repeat the same procedure using the total derivative and again using Eq. (24) we derive

C22 $$\begin{aligned} \frac{dn_{\text {hot}}}{\text {d}L}&=-\frac{n_{\text {hot}}}{\alpha L}\exp \Bigg (\beta _{\text {hot}}^2 \varsigma _{\text {hot}} \Bigg (1-\exp \Bigg (-\frac{\breve{k}_0}{\alpha }G\nonumber \\&\quad \times \Bigg (\frac{1}{L}\Bigg )\Bigg )\Bigg ) s_{H,e}^2\Bigg ). \end{aligned}$$

Continuing in the same manner, by once again integrating we calculate,

C23 $$\begin{aligned}&n_{\text {hot}}=\exp \left( -\frac{1}{\alpha }G_{\text {hot}}\left( \frac{1}{L}\right) \right) \ \text {with} \end{aligned}$$

C24 $$\begin{aligned}&G_{\text {hot}}(y)=\int ^{1}_y \frac{\exp \left( \beta _{\text {hot}}^2 \varsigma _{\text {hot}} \left( 1- \exp \left( -\frac{\breve{k}_0}{\alpha }G(\hat{y})\right) \right) s_{H,e}^2\right) }{\hat{y}} \ \text {d}\hat{y}. \end{aligned}$$

## Appendix D: Solving the cellulose angle equation

To find the angle cellulose makes to the horizontal, $\theta$, we solve Eq. (28), by first re-expressing left-hand side as the total derivative to get

D25 $$\begin{aligned} \frac{\text {d}\theta }{\text {d}t}=\alpha \sin \theta \cos \theta \ \text {on} \ \frac{\text {d}y}{\text {d}t}=-\alpha y. \end{aligned}$$

Beginning with the second group of fibres deposited at $y=1$ for $t=\tau >0$ with angle $\theta (\tau ,1)=\theta _0$, we consider $\bar{t}=t-\tau$. Consequently on integrating $\frac{\text {d}\theta }{d \bar{t}}=\alpha \sin (\theta )\cos (\theta )$, we get

D26 $$\begin{aligned} \int ^{\theta }_{\theta _0}\frac{1}{\sin \theta '\cos \theta '}\ \text {d}\theta '=\int ^{\bar{t}}_{0}\alpha \ \text {d}\tau '. \end{aligned}$$

Making the substitution $v=\tan (\theta )$ we calculate,

D27 $$\begin{aligned} \int ^v_{v_0}\frac{1}{v'}\ dv'=\alpha \bar{t} , \end{aligned}$$

which we integrate to find,

D28 $$\begin{aligned} \tan \theta (\bar{t},y)=\tan \theta _0\exp \left( \alpha \bar{t}\right) . \end{aligned}$$

Knowing $y=\exp \left( -\alpha \bar{t}\right)$ using $y=1$ at $\bar{t}=0$ we get

D29 $$\begin{aligned} \theta (t,y)=\arctan \left( \frac{\tan \theta _0}{y} \right) \ \text {for} \ e^{-\alpha t} < y \le 1.\qquad \end{aligned}$$

Following the same procedure for the first group of crosslinks present at $t=0$ positioned at $y=y_i$ with angle $\theta (t,y_i)=\theta _0$ we derive,

D30 $$\begin{aligned} \theta (t,y)=\arctan \left( \tan \theta _0 e^{\alpha t}\right) \ \text {for} \ 0\le y \le e^{-\alpha t}. \end{aligned}$$

For the cellulose length we once again use the transformed co-ordinate $\bar{t}$ to get,

D31 $$\begin{aligned} \frac{1}{L_C}\frac{d L_C}{\text {d}\bar{t}}=\alpha \sin ^2(\theta (\bar{t},y)), \end{aligned}$$

using the separation of variables we get

D32 $$\begin{aligned} \ln (L_C)=\int ^{\bar{t}}_0\alpha \sin (\theta (\hat{t},y)) \text {d}\hat{t}. \end{aligned}$$

For our different domains we then get,

D33 $$\begin{aligned} L_C(t)=\exp \left( \alpha \int ^{\bar{t}}_0 \sin ^2\theta (\hat{t}) \ \text {d}\hat{t} \right) \ \text {for} \ 0\le y \le e^{-\alpha t} , \end{aligned}$$

and

D34 $$\begin{aligned} L_C(y)=\exp \left( \int ^{1}_y \frac{\sin ^2\theta (\hat{y})}{\hat{y}} \ \text {d}\hat{y} \right) \ \text {for} \ e^{-\alpha t} < y \le 1. \end{aligned}$$

## Appendix E: Cel12A cellulose action

To model Cel12A’s digestion action on cellulose, we assume it reduces the cellulose density $\rho$. For simplicity, this will take the form.

E35 $$\begin{aligned} \frac{\partial \rho ^*}{\partial t^*}=C^*\rho ^*\left( 1-\frac{\rho ^*}{\rho ^*_1}\right) \end{aligned}$$

with $\rho ^*(h^*,\tau ^*)=\rho ^*(y^*,0)=\rho _0^*$ where $C^*$ is Cel12A’s action rate and is proportional to $\beta _{\text {hot}}$, meaning $C^*=\tilde{C}^* \beta _{\text {hot}}$. We take this form to ensure that $\rho ^*\ne 0$ and to limit digestion. In the non-dimensional system, the above becomes

E36 $$\begin{aligned} \frac{\partial \rho }{\partial t}=C\rho \left( 1-\mathcal {N}\rho \right) \end{aligned}$$

with $\rho (1,\tau )=\rho (y,0)=1$ and $\mathcal {N}=\frac{\rho _0^*}{\rho _1^*}$. The non-dimensionalisation now changes to $\rho ^*=\rho _0^*\rho$, $C^*=k^*_{0,\text {hot}}C$, $\tilde{C}^*=k^*_{0,\text {hot}}\tilde{C}$, $\mathcal {E}^*=\nu _C^* h^* \rho ^*_0$ and $a_2=\frac{n^*_0n^*_{0,\text {hot}}\nu ^*_{H}}{\rho _0^*\nu _C^*}$ which will have the same value as $a_2$ shown in Table 1. Equation E36 can be solved to get

E37 $$\begin{aligned} \rho =\frac{1}{\mathcal {N}+\left( 1-\mathcal {N}e^{-Ct}\right) } =\frac{1}{\mathcal {N}+\left( 1-\mathcal {N}y^{\frac{C}{\alpha }}\right) }. \end{aligned}$$

The steady-state stress result then becomes

E38 $$\begin{aligned} \Sigma ^{\infty }&=\int ^1_{\epsilon }\rho \left( 1+a_1\exp \left( -\frac{1}{\alpha }G_{\text {hot}} \left( y\right) \right) \right) \nonumber \\&\quad \left( \exp \left( \int ^1_y \frac{\sin ^2\theta (\hat{y})}{y} \text {d}\hat{y}\right) -1\right) \nonumber \\&\quad \times \sin \left( \arctan \left( \frac{\tan (\theta _0)}{y}\right) \right) \nonumber \\&+a_2\exp \left( -\frac{k_0}{k_{0,hot}}\frac{1}{\alpha }G\left( y\right) \right) \exp \left( -\frac{1}{\alpha }G_{\text {hot}}\left( y\right) \right) \nonumber \\&\qquad \qquad \qquad \sigma _H(y) \ \text {d}y +\Gamma \alpha . \end{aligned}$$

Figure 10 shows the effect of Cel12A additional cellulose digestion where it simply decreases the stress resultant (significantly) more when compared to Fig. 5 where it just affects the hotspots.

## Appendix F: Asymptotic expansion

Beginning with the asymptotic expansion of *G*, we consider the two regions, $\gamma \ll y \le 1$ and $\epsilon \le y \ll \gamma$. In considering the first case, we know that $\left( \frac{1}{z}-z^{\frac{\omega }{\alpha }}\right) =\frac{1}{z}\left( 1-z^{\frac{\omega }{\alpha }+1}\right)$ and as $0< z\le 1$, and $\frac{\omega }{\alpha }>0$ we have $0< z^{\frac{\omega }{\alpha }+1} \le 1$ which implies the following bound, $0 <\left( 1-z^{\frac{\omega }{\alpha }+1}\right) \le 1$. Using this we find,

F39 $$\begin{aligned} G(y)&=\int ^1_y \frac{\exp \left( \gamma ^2\breve{\alpha }^2\left( \frac{1}{z}-z^{\frac{\omega }{\alpha }}\right) ^2\right) }{z} \ dz \nonumber \\&\le \int ^1_y \frac{\exp \left( \breve{\alpha }^2\left( \frac{\gamma }{z}\right) ^2\right) }{z} \ dz, \end{aligned}$$

and as $\frac{\gamma }{y}\ll 1$, we then have

F40 $$\begin{aligned} G(y)\approx \int ^1_y \frac{1}{z} \ dz=\ln \left( \frac{1}{y}\right) . \end{aligned}$$

Alternatively, for the second case, $\epsilon \le y \ll \gamma$ we have $\frac{y}{\gamma }\ll 1$ where we can once again use the upper bound of, $\left( 1-z^{\frac{\omega }{\alpha }+1}\right) \le 1$ to get,

F41 $$\begin{aligned} G(y)\le \int ^1_y \frac{\exp \left( \breve{\alpha }^2\frac{\gamma ^2}{z^2}\right) }{z} \ dz. \end{aligned}$$

Using integration by parts and knowing that $\frac{d}{dz}\left( \exp \left( \breve{\alpha }^2\frac{\gamma ^2}{z^2}\right) \right) =-\frac{2\gamma ^2\breve{\alpha }^2}{z^3}\exp \left( \breve{\alpha }^2\frac{\gamma }{z^2}\right)$, we calculate,

F42 $$\begin{aligned} G(y)&\le \int ^1_y -\frac{z^2}{2\breve{\alpha }^2\gamma ^2}\frac{d}{dz} \left( \exp \left( \breve{\alpha }^2\frac{\gamma ^2}{z^2}\right) \right) \ dz\nonumber \\&= \left[ -\frac{z^2}{2\breve{\alpha }^2 \gamma ^2} \exp \left( \breve{\alpha }^2\frac{\gamma ^2}{z^2}\right) \right] ^1_y + \int ^1_y\frac{z}{\breve{\alpha }^2\gamma ^2}\exp \left( \breve{\alpha }^2\frac{\gamma ^2}{z^2}\right) \ dz \nonumber \\&=-\frac{1}{2\breve{\alpha }^2\gamma ^2}\exp (\gamma ^2\breve{\alpha }^2) + \frac{y^2}{2\breve{\alpha }^2 \gamma ^2}\exp \left( \breve{\alpha }^2\frac{\gamma ^2}{y^2}\right) \nonumber \\&-\int ^1_y \frac{z^4}{2\breve{\alpha }^4\gamma ^4}\frac{d}{dz} \left( \exp \left( \breve{\alpha }^2\frac{\gamma ^2}{z^2}\right) \right) \ dz. \end{aligned}$$

Using the fact that $\gamma$ is small, we can ignore the first term, and if we then repeatedly integrate by parts, we can get the sequence

F43 $$\begin{aligned} G(y)=\frac{y^2}{2 \breve{\alpha }^2\gamma ^2}\exp \left( \breve{\alpha }^2\frac{\gamma ^2}{y^2}\right) + \frac{y^4}{2\breve{\alpha }^2 \gamma ^4}\exp \left( \breve{\alpha }^2\frac{\gamma ^2}{y^2}\right) +\dots . \end{aligned}$$

As $\frac{y}{\gamma }\ll 1$ meaning $\frac{y^2}{\gamma ^2}>\frac{y^k}{\gamma ^k}$ where $k=4,6,8,\dots$ we then have,

F44 $$\begin{aligned} G(y)\approx \frac{y^2}{2\breve{\alpha }^2 \gamma ^2}\exp \left( \breve{\alpha }^2\frac{\gamma ^2}{y^2}\right) +o\left( \frac{y^2}{2\breve{\alpha }^2 \gamma ^2}\exp \left( \breve{\alpha }^2\frac{\gamma ^2}{y^2}\right) \right) . \end{aligned}$$

From Eq. (38), the asymptotic approximation of *n* is then,

F45 $$\begin{aligned} n= {\left\{ \begin{array}{ll} \exp \left( -\frac{\breve{k}_0}{\alpha }\frac{y^2}{2\breve{\alpha }^2\gamma ^2} e^{\breve{\alpha }^2\frac{\gamma ^2}{y^2}}\right) , \quad \epsilon \le y \ll \gamma \\ y^{\frac{\breve{k}_0}{\alpha }}, \hspace{34mm} \gamma \ll y \le 1. \end{array}\right. } \end{aligned}$$

Writing $\breve{k}_0=\frac{k_{0}}{k_{0,\text {hot}}}$, we now calculate the asymptotic expansion of $G_{\text {hot}}$. To find the approximation, we begin in the region $\gamma \ll y \le 1$ and $\beta _{\text {hot}}\ll y \le 1$, where upon using the expansion of *n* in this same region (Eq. (47)) and the upper bound of the extension ($\left( 1-z^{\frac{\omega }{\alpha }+1}\right) <1$) we get

F46 $$\begin{aligned} G_{\text {hot}}(y)&=\int ^1_y \frac{\exp \left( \beta _{\text {hot}}^2 \breve{\alpha }^2 \left( 1-z^{\frac{\breve{k}_0}{\alpha }}\right) \left( \frac{1}{z}-z^{\frac{\omega }{\alpha }}\right) ^2\right) }{z} \ \text {d}y \end{aligned}$$

F47 $$\begin{aligned}&\le \int ^1_y \frac{\exp \left( \beta _{\text {hot}}^2 \breve{\alpha }^2 \frac{1}{z^2}\left( 1-z^{\frac{\breve{k}_0}{\alpha }}\right) \right) }{z} \ \text {d}y. \end{aligned}$$

To find the leading-order terms, we Taylor expand the exponential around $\beta _{\text {hot}}=0$ to find,

F48 $$\begin{aligned}&=\int ^1_y \frac{1}{z}\Bigg ( 1+\frac{\beta _{\text {hot}}^2\breve{\alpha }^2 }{z^2} \left( 1-z^{\frac{\breve{k}_0}{\alpha }}\right) \nonumber \\&\quad +\frac{\beta _{\text {hot}}^4\breve{\alpha }^4 }{2z^4}\left( 1-z^{\frac{\breve{k}_0}{\alpha }}\right) ^2+\dots \Bigg ) \ \text {d}y. \end{aligned}$$

Lets consider any term in the series, $\left( 1-z^{\frac{\breve{k}_0}{\alpha }}\right) ^i$, with $i\in {\mathbb {N}}/\{0\}$. We want to find the biggest term in this bracket. We begin by assuming that 1 is the biggest term and using the fact that $\frac{\breve{k}_0}{\alpha }>0$ and $0< z\le 1$ we have,

F49 $$\begin{aligned} \Vert 1\Vert \ge \Vert z^{\frac{\breve{k}_0}{\alpha }}\Vert \Rightarrow 1\ge z^{\frac{\breve{k}_0}{\alpha }} \Rightarrow 1\ge z . \end{aligned}$$

So 1 is indeed the bigger term meaning $1-z^{\frac{\breve{k}_0}{\alpha }} \le 1$. Now we need to show that the 1st term in the integral (Eq. (F48)) is greater than $\frac{\beta _{\text {hot}}^{2i}\breve{\alpha }^{2i}}{i!z^{2i}}\left( 1-z^{\frac{\breve{k}_0}{\alpha }}\right) ^i$ for each $i\in {\mathbb {N}}/\{0\}$. From the workings above, we know the ith term is less than, $\frac{\beta _{\text {hot}}^{2i}\breve{\alpha }^{2i}}{i!z^{2i}}$ so on assuming the first term, 1 is bigger than the other terms for any *i*, we have $\frac{\beta _{\text {hot}}^{2i}\breve{\alpha }^{2i}}{i!z^{2i}}<1\Rightarrow \left( \frac{\beta _{\text {hot}}^{2}\breve{\alpha }^2}{i!z^2}\right) ^i<1\Rightarrow \frac{\beta _{\text {hot}}^2}{z^2}<\frac{i!}{\breve{\alpha }^2}$ and since $\frac{\beta _{\text {hot}}}{z}\ll 1$, we now know 1 is indeed the biggest term in the sequence. Therefore, we can ignore the higher-order terms in Eq. (F48) to get

F50 $$\begin{aligned} G_{\text {hot}}(y)\approx \int ^1_y \frac{1}{z} \ dz =\ln \left( \frac{1}{y}\right) . \end{aligned}$$

Deriving the expansion for $\epsilon <y\ll \beta _{\text {hot}}$ and $\epsilon <y\ll \gamma$ we have that,

F51 $$\begin{aligned}&G_{\text {hot}}(y)\nonumber \\&=\int ^1_y \frac{\exp \left( \beta _{\text {hot}}^2\breve{\alpha }^2\left( 1-\exp \left( -\frac{\breve{k}_0}{\alpha }\frac{y^2}{2\breve{\alpha }^2\beta _{\text {hot}}^2} e^{\frac{\beta _{\text {hot}}^2}{y^2}}\right) \right) \left( \frac{1}{z}-z^{\frac{\omega }{\alpha }}\right) ^2\right) }{z} \ \text {d}y \nonumber \\&\le \int ^1_y \frac{\exp \left( \beta _{\text {hot}}^2\breve{\alpha }^2\frac{1}{z^2}\right) }{z} \ \text {d}y, \end{aligned}$$

by using the upper bound of the extension again and the fact that if $\frac{y}{\beta _{\text {hot}}}\ll 1$ then $e^{\frac{\beta _{\text {hot}}^2}{y^2}}\gg 1$ and consequently $\exp \left( -\frac{\breve{k}_0}{\alpha }\frac{y^2}{2\breve{\alpha }^2\beta _{\text {hot}}^2}e^{\frac{\beta _{\text {hot}}^2}{y^2}} \right) \ll 1$. We can repeat the procedure of solving Eq. (F41) to derive

F52 $$\begin{aligned} G_{\text {hot}}(y)=\frac{y^2}{2 \breve{\alpha }^2\beta _{\text {hot}}^2}\exp \left( \breve{\alpha }^2 \frac{\beta _{\text {hot}}^2}{y^2}\right) +o\left( \frac{y^4}{\gamma ^4}\right) . \end{aligned}$$

The case $\beta _{\text {hot}}<y<\gamma$ is a hybrid of the two methods. You can ignore the exponential term in a similar way to (F51) and follow the same derivation for *G* in Eq. (F40) to derive $G_{\text {hot}}=\ln \left( \frac{1}{y}\right)$. Collecting the results together we get,

F53 $$\begin{aligned} n_{\text {hot}}= {\left\{ \begin{array}{ll} \exp \left( -\frac{1}{\alpha }\frac{y^2}{2\breve{\alpha }^2\beta _{\text {hot}}^2} e^{\breve{\alpha }\frac{\beta _{\text {hot}}^2}{y^2}}\right) , \quad \epsilon \le y \ll \beta _{\text {hot}} \\ y^{\frac{1}{\alpha }}, \hspace{33mm}\beta _{\text {hot}} \ll y \le 1 . \end{array}\right. } \end{aligned}$$

Notice that the simplification of $n_{\text {hot}}$ is not dependant on *n*.

We need to know exactly where the different asymptotic expansions in the different regions can be applied. Starting with *n*, the first boundary region, $\epsilon <y \ll \beta _{\text {hot}}$, applies when *n* has rapidly decreased. So if we find the value for *y* for when this happens, we can get out a critical value for when the different expansions can be used. To find this value, we use the derivative of the dominate term of the exponent of *n* which is $\frac{\breve{k}_0}{\alpha }\exp \left( \frac{\gamma ^2\breve{\alpha }}{y^2}\right)$ and find the value when it exceeds a certain number, as when this rapidly increases, *n* rapidly decreases. This value is then,

F54 $$\begin{aligned} \chi =\sqrt{\frac{\gamma ^2\breve{\alpha }^2}{\ln \left( \frac{\alpha }{\breve{k_0}}Q\right) }}, \end{aligned}$$

where *Q* is a threshold value of our choice. The threshold value for $n_{\text {hot}}$, similarly is,

F55 $$\begin{aligned} \chi _{\text {hot}}=\sqrt{\frac{\beta _{\text {hot}}^2\breve{\alpha }^2}{\ln \left( \alpha Q\right) }}. \end{aligned}$$

To be able to solve the limit stress resultant, we need to also approximate the trigonometric term present, $\sin \left( \arctan \left( \tan (\theta _0)\frac{1}{y}\right) \right)$. Note for $\tan \theta _0<y\le 1$ we have $\frac{\tan \theta _0}{y}<1$. Using the Taylor expansions on both $\arctan$ and $\sin$ and ignoring higher-order terms, we can derive

F56 $$\begin{aligned}&\sin \left( \arctan \left( \tan (\theta _0)\frac{1}{y} \right) \right) \end{aligned}$$

F57 $$\begin{aligned}&\quad =\sin \left( \frac{\tan \theta _0}{y} -\frac{\tan ^3\theta _0}{3y^3}+\frac{\tan ^5\theta _0}{5y^5}-\frac{\tan ^7\theta _0}{7y^7}+\dots \right) \end{aligned}$$

F58 $$\begin{aligned}&\quad =\left( \frac{\tan \theta _0}{y}-\frac{\tan ^3\theta _0}{3y^3}+\frac{\tan ^5\theta _0}{5y^5} -\frac{\tan ^7\theta _0}{7y^7}+\dots \right) \end{aligned}$$

F59 $$\begin{aligned}&\qquad -\frac{1}{3!}\left( \frac{\tan \theta _0}{y} -\frac{\tan ^3\theta _0}{3y^3}+\frac{\tan ^5\theta _0}{5y^5}-\frac{\tan ^7\theta _0}{7y^7}+\dots \right) ^3 \end{aligned}$$

F60 $$\begin{aligned}&\qquad +\frac{1}{5!}\left( \frac{\tan \theta _0}{y}-\frac{\tan ^3\theta _0}{3y^3}+\frac{\tan ^5\theta _0}{5y^5} -\frac{\tan ^7\theta _0}{7y^7}+\dots \right) ^5 \end{aligned}$$

F61 $$\begin{aligned}&\qquad -\frac{1}{7!}\left( \frac{\tan \theta _0}{y} -\frac{\tan ^3\theta _0}{3y^3}+\frac{\tan ^5\theta _0}{5y^5}-\frac{\tan ^7\theta _0}{7y^7}+\dots \right) ^7+\dots \end{aligned}$$

F62 $$\begin{aligned}&\quad =\frac{\tan \theta _0}{y}-\frac{1}{2}\frac{\tan ^3\theta _0}{y^3} +\frac{3}{8}\frac{\tan ^5\theta _0}{y^5} -\frac{5}{16}\frac{\tan ^7\theta _0}{y^7}\end{aligned}$$

F63 $$\begin{aligned}&\qquad + o\left( \frac{\tan ^9\theta _0}{y^9}\right) . \end{aligned}$$

For the case $\gamma<y<\tan \theta _0$ we use the relation, $\sin \left( \arctan \left( \tan \theta _0\frac{1}{y}\right) \right) =\frac{\tan \theta _0}{\sqrt{\tan ^2\theta _0+y^2}}$. Noting that $\frac{y}{\tan \theta _0}<1$ and using the Taylor expansion around $y=0$ we find,

F64 $$\begin{aligned} \frac{\tan \theta _0}{\sqrt{\tan ^2\theta _0+y^2}}&=1-\frac{y^2}{2\tan ^2\theta _0} +\frac{3y^4}{8\tan ^4\theta _0}-\frac{5y^6}{16\tan ^6\theta _0}\end{aligned}$$

F65 $$\begin{aligned}&+o\left( \frac{y^8}{\tan ^8\theta _0}\right) . \end{aligned}$$

Both of these approximations lose accuracy around $y=\tan _0$, we therefore repeat the procedure above but now Taylor expand around $y=\tan _0$ to get,

F66 $$\begin{aligned} \displaystyle \frac{\tan \theta _0}{\sqrt{\tan ^2\theta _0+y^2}}&=\frac{1}{\sqrt{2}} -\frac{y-\tan \theta _0}{2\sqrt{2}\tan \theta _0}+\frac{(y-\tan \theta )^2}{8\sqrt{2}\tan ^2\theta _0}\nonumber \\&\displaystyle \quad +\frac{(y-\tan \theta )^3}{16\sqrt{2}\tan ^3\theta _0}\nonumber \\&\displaystyle \quad -\frac{13 (y - \tan \theta _0)^4}{128 \sqrt{2}\tan \theta _0^4} +o\left( \frac{y^5}{\tan ^5\theta _0}\right) . \end{aligned}$$

The approximation of the trigonometric term is then defined by three different expansions in three different regions, such that,

F67

Where $\frac{\tan \theta _0}{2}$ and $\tan \theta _0+\delta$ are interval values of our choosing to ensure accuracy. We can use this expression for the cellulose angle to find the leading terms that dominate the celluloses length’s, $L_C$ behaviour. Substituting in (51), we get

F68

where $\sim$ is used for simplicity to represent the integrand that appears in the previous line for the same integral region. Upon evaluating our integral, we find,

F69

where

F70 $$\begin{aligned} B_1(y)&=-\frac{\tan ^2\theta _0}{2\hat{y}^2}+\frac{\tan ^4\theta _0}{4\hat{y}^4}\nonumber \\&\quad -\frac{7\tan ^6\theta _0}{54\hat{y}^6}+\frac{19\tan ^8\theta _0}{576\hat{y}^8} -\frac{361\tan ^{10}\theta _0}{51840\hat{y}^{10}}, \end{aligned}$$

F71 $$\begin{aligned} B_2(y)&=\frac{34969}{32768}\log (y)- \frac{935y}{4096\tan \theta _0} -\frac{7941y^2}{16384\tan ^2\theta _0}\nonumber \\&\quad +\frac{3235y^3}{12288\tan ^3\theta _0} \nonumber \\&\quad +\frac{67y^4}{65536\tan ^4\theta _0}-\frac{245y^5}{4096\tan ^5\theta _0} +\frac{1459y^6}{49152\tan ^6\theta _0} \nonumber \\&\quad -\frac{195y^7}{28672\tan ^7\theta _0} +\frac{169y^8}{262144\tan ^8\theta _0}, \end{aligned}$$

F72 $$\begin{aligned} B_3(y)&=\log (y)- \frac{y^2}{2\tan ^2\theta _0}+\frac{y^4}{4\tan ^4\theta _0} \nonumber \\&\quad -\frac{y^6}{6\tan ^6\theta _0}+\frac{29y^8}{512\tan ^8\theta _0}-\frac{3y^{10}}{128\tan ^{10}\theta _0}\nonumber \\&\quad +\frac{25y^{12}}{3072\tan ^{12}\theta _0}. \end{aligned}$$

When we ignore the higher-order terms and expanding Taylor expanding the exponential we get the final form as,

F73 $$\begin{aligned}&L_C(y)\nonumber \\&={\left\{ \begin{array}{ll} b_1\left( 1+\frac{\tan ^2\theta _0}{2\hat{y}^2}\right) , \hspace{18mm} \tan \theta _0+\delta<y\le 1, \\ \frac{b_2}{y^{\frac{34969}{32768}}} (1-H(y)+\frac{H(y)^2}{2}), \hspace{3mm} \frac{\tan \theta _0}{2} <y\le \tan \theta _0+\delta , \\ \frac{b_3}{y}\left( 1+\frac{y^2}{2\tan ^2\theta _0}\right) , \hspace{17mm} \epsilon \le y\le \frac{\tan \theta _0}{2}, \end{array}\right. } \end{aligned}$$

where

F74 $$\begin{aligned}&b_1=e^{B_1(1)}, \end{aligned}$$

F75 $$\begin{aligned}&b_2=e^{B_1(1)}e^{-B_1((\tan \theta _0+\delta ))}e^{B_2(\tan \theta _0+\delta )},\end{aligned}$$

F76 $$\begin{aligned}&b_3=e^{B_1(1)}e^{-B_1((\tan \theta _0+\delta ))}e^{B_2(\tan \theta _0+\delta )} e^{-B_2(\frac{\tan \theta _0}{2})}e^{B_3(\frac{\tan \theta _0}{2})}, \nonumber \\&\ \text {and} \end{aligned}$$

F77 $$\begin{aligned}&H(y)=B_2(2)-\frac{34969}{32768}\log (y) \end{aligned}$$

**Table 2:** Asymptotic expansion constants for $\Sigma _1^\infty$

Constant	Value	Constant	Value
$c_{1}$	$\frac{305\,b_{3}}{196608}$	$c_{2}$	$\epsilon$
$c_{3}$	$\frac{766290879471899\,\tan \theta _0}{118219490218475520}$	$c_{4}$	$b_{3}\,\ln \left( \frac{\tan \theta _0}{2}\right)$
$c_{5}$	$\tan \theta _0\ln \left( \delta +\tan \theta _0\right)$	$c_{5}$	$-b_{1}\,\tan \theta _0\ln \left( \delta +\tan \theta _0\right)$
$c_{6}$	$-\frac{150077200448881\,b_{2}}{9758165696512\,{\left( \delta +\tan \theta _0\right) }^{2201/32768}}$	$c_{7}$	$-b_{3}\,\ln \left( \epsilon \right)$
$c_{7}$	$-\frac{b_{1}\,{\tan \theta _0}^5}{32}$	$c_{8}$	$\frac{b_{1}\,{\tan \theta _0}^7}{48}$
$c_{9}$	$\frac{5\,b_{1}\,{\tan \theta _0}^9}{256}$	$c_{10}$	$-\frac{{\tan \theta _0}^3}{4}$
$c_{11}$	$\frac{3\,{\tan \theta _0}^5}{32}$	$c_{12}$	$-\frac{5\,{\tan \theta _0}^7}{96}$
$c_{13}$	$\frac{-3\,{\tan \theta _0}^5}{32\,{\left( \delta +\tan \theta _0\right) }^4}$	$c_{14}$	$\frac{b_{1}\,{\tan \theta _0}^5}{32\,{\left( \delta +\tan \theta _0\right) }^4}$
$c_{15}$	$\frac{-2\,b_{1}\,{\tan \theta _0}^7}{96\,{\left( \delta +\tan \theta _0\right) }^6}$	$c_{16}$	$\frac{5\,{\tan \theta _0}^7}{96\,{\left( \delta +\tan \theta _0\right) }^6}$
$c_{17}$	$\frac{-\frac{5\,b_{1}\,{\tan \theta _0}^9}{8}}{32\,{\left( \delta +\tan \theta _0\right) }^8}$	$c_{18}$	$\frac{8\,{\tan \theta _0}^3\,{\left( \delta +\tan \theta _0\right) }^6}{32\,{\left( \delta +\tan \theta _0\right) }^8}$
$c_{19}$	$\frac{150077200448881\,b_{2}}{9758165696512\,{\left( \frac{\tan \theta _0}{2}\right) }^{2201/32768}}$	$c_{20}$	$-\frac{187\,\sqrt{2}\,\left( \delta +\tan \theta _0\right) }{256}$
$c_{21}$	$-\frac{\epsilon ^3}{6\,{\tan \theta _0}^2}$	$c_{22}$	$\frac{3\,\epsilon ^5}{40\,{\tan \theta _0}^4}$
$c_{23}$	$-\frac{5\,\epsilon ^7}{112\,{\tan \theta _0}^6}$	$c_{24}$	$\frac{5\,\sqrt{2}\,{\left( \delta +\tan \theta _0\right) }^2}{128\,\tan \theta _0}$
$c_{25}$	$\frac{43\,\sqrt{2}\,{\left( \delta +\tan \theta _0\right) }^3}{384\,{\tan \theta _0}^2}$	$c_{26}$	$-\frac{15\,\sqrt{2}\,{\left( \delta +\tan \theta _0\right) }^4}{256\,{\tan \theta _0}^3}$
$c_{27}$	$\frac{13\,\sqrt{2}\,{\left( \delta +\tan \theta _0\right) }^5}{1280\,{\tan \theta _0}^4}$	$c_{28}$	$\frac{6020549791152521\,b_{2}\,{\left( \delta +\tan \theta _0\right) }^{30567/32768}}{44811695901638656\,\tan \theta _0}$
$c_{29}$	$\frac{7868723041398407\,b_{2}\,{\left( \delta +\tan \theta _0\right) }^{63335/32768}}{557100551561543680\,{\tan \theta _0}^2}$	$c_{30}$	$\frac{4402425010544735\,b_{2}\,{\left( \delta +\tan \theta _0\right) }^{96103/32768}}{1449138733222723584\,{\tan \theta _0}^3}$
$c_{31}$	$-\frac{81932115935621\,b_{2}\,{\left( \delta +\tan \theta _0\right) }^{128871/32768}}{1967988374765568\,{\tan \theta _0}^4}$	$c_{32}$	$\frac{2682583518496071\,b_{2}\,{\left( \delta +\tan \theta _0\right) }^{161639/32768}}{88861980001042432\,{\tan \theta _0}^5}$
$c_{33}$	$-\frac{7116713214246575\,b_{2}\,{\left( \delta +\tan \theta _0\right) }^{194407/32768}}{480943703297359872\,{\tan \theta _0}^6}$	$c_{34}$	$\frac{89993661700897\,b_{2}\,{\left( \delta +\tan \theta _0\right) }^{227175/32768}}{6917027650338816\,{\tan \theta _0}^7}$
$c_{35}$	$-\frac{1390888037066579\,b_{2}\,{\left( \delta +\tan \theta _0\right) }^{259943/32768}}{160768322470674432\,{\tan \theta _0}^8}$	$c_{36}$	$\frac{813773447008073\,b_{2}\,{\left( \delta +\tan \theta _0\right) }^{292711/32768}}{633620822778445824\,{\tan \theta _0}^9}$
$c_{37}$	$\frac{280915086652931\,b_{2}\,{\left( \delta +\tan \theta _0\right) }^{325479/32768}}{134200479411339264\,{\tan \theta _0}^{10}}$	$c_{38}$	$-\frac{5278606494654121\,b_{2}\,{\left( \delta +\tan \theta _0\right) }^{358247/32768}}{3101936844162465792\,{\tan \theta _0}^{11}}$
$c_{39}$	$\frac{3916342628302019\,b_{2}\,{\left( \delta +\tan \theta _0\right) }^{391015/32768}}{6771327241373614080\,{\tan \theta _0}^{12}}$	$c_{40}$	$-\frac{4112068129940143\,b_{2}\,{\left( \delta +\tan \theta _0\right) }^{423783/32768}}{117420492710756745216\,{\tan \theta _0}^{13}}$
$c_{41}$	$-\frac{1121251467598159\,b_{2}\,{\left( \delta +\tan \theta _0\right) }^{456551/32768}}{18447880144098951168\,{\tan \theta _0}^{14}}$	$c_{42}$	$\frac{1772224420073771\,b_{2}\,{\left( \delta +\tan \theta _0\right) }^{489319/32768}}{52725169158789005312\,{\tan \theta _0}^{15}}$
$c_{43}$	$-\frac{1694538362442335\,b_{2}\,{\left( \delta +\tan \theta _0\right) }^{522087/32768}}{168767973799942422528\,{\tan \theta _0}^{16}}$	$c_{44}$	$\frac{94162698713311\,b_{2}\,{\left( \delta +\tan \theta _0\right) }^{554855/32768}}{47829450699604754432\,{\tan \theta _0}^{17}}$
$c_{45}$	$-\frac{388717606998977\,b_{2}\,{\left( \delta +\tan \theta _0\right) }^{587623/32768}}{1519623251576745885696\,{\tan \theta _0}^{18}}$	$c_{46}$	$\frac{1057140962472757\,b_{2}\,{\left( \delta +\tan \theta _0\right) }^{620391/32768}}{52154529959914841833472\,{\tan \theta _0}^{19}}$
$c_{47}$	$-\frac{2775672680954111\,b_{2}\,{\left( \delta +\tan \theta _0\right) }^{653159/32768}}{3707279049981556007370752\,{\tan \theta _0}^{20}}$	$c_{48}$	$-\frac{b_{3}\,\epsilon ^4}{32\,{\tan \theta _0}^4}$
$c_{49}$	$\frac{b_{3}\,\epsilon ^6}{48\,{\tan \theta _0}^6}$	$c_{50}$	$\frac{5\,b_{3}\,\epsilon ^8}{256\,{\tan \theta _0}^8}$
$c_{51}$	$-\frac{6020549791152521\,b_{2}\,{\left( \frac{\tan \theta _0}{2}\right) }^{30567/32768}}{44811695901638656\,\tan \theta _0}$	$c_{52}$	$-\frac{7868723041398407\,b_{2}\,{\left( \frac{\tan \theta _0}{2}\right) }^{63335/32768}}{557100551561543680\,{\tan \theta _0}^2}$
$c_{53}$	$-\frac{4402425010544735\,b_{2}\,{\left( \frac{\tan \theta _0}{2}\right) }^{96103/32768}}{1449138733222723584\,{\tan \theta _0}^3}$	$c_{54}$	$\frac{81932115935621\,b_{2}\,{\left( \frac{\tan \theta _0}{2}\right) }^{128871/32768}}{1967988374765568\,{\tan \theta _0}^4}$
$c_{55}$	$-\frac{2682583518496071\,b_{2}\,{\left( \frac{\tan \theta _0}{2}\right) }^{161639/32768}}{88861980001042432\,{\tan \theta _0}^5}$	$c_{56}$	$\frac{7116713214246575\,b_{2}\,{\left( \frac{\tan \theta _0}{2}\right) }^{194407/32768}}{480943703297359872\,{\tan \theta _0}^6}$
$c_{57}$	$-\frac{89993661700897\,b_{2}\,{\left( \frac{\tan \theta _0}{2}\right) }^{227175/32768}}{6917027650338816\,{\tan \theta _0}^7}$	$c_{58}$	$\frac{1390888037066579\,b_{2}\,{\left( \frac{\tan \theta _0}{2}\right) }^{259943/32768}}{160768322470674432\,{\tan \theta _0}^8}$
$c_{59}$	$-\frac{813773447008073\,b_{2}\,{\left( \frac{\tan \theta _0}{2}\right) }^{292711/32768}}{633620822778445824\,{\tan \theta _0}^9}$	$c_{60}$	$-\frac{280915086652931\,b_{2}\,{\left( \frac{\tan \theta _0}{2}\right) }^{325479/32768}}{134200479411339264\,{\tan \theta _0}^{10}}$
$c_{61}$	$\frac{5278606494654121\,b_{2}\,{\left( \frac{\tan \theta _0}{2}\right) }^{358247/32768}}{3101936844162465792\,{\tan \theta _0}^{11}}$	$c_{62}$	$-\frac{3916342628302019\,b_{2}\,{\left( \frac{\tan \theta _0}{2}\right) }^{391015/32768}}{6771327241373614080\,{\tan \theta _0}^{12}}$
$c_{63}$	$\frac{4112068129940143\,b_{2}\,{\left( \frac{\tan \theta _0}{2}\right) }^{423783/32768}}{117420492710756745216\,{\tan \theta _0}^{13}}$	$c_{64}$	$\frac{1121251467598159\,b_{2}\,{\left( \frac{\tan \theta _0}{2}\right) }^{456551/32768}}{18447880144098951168\,{\tan \theta _0}^{14}}$
$c_{65}$	$-\frac{1772224420073771\,b_{2}\,{\left( \frac{\tan \theta _0}{2}\right) }^{489319/32768}}{52725169158789005312\,{\tan \theta _0}^{15}}$	$c_{66}$	$\frac{1694538362442335\,b_{2}\,{\left( \frac{\tan \theta _0}{2}\right) }^{522087/32768}}{168767973799942422528\,{\tan \theta _0}^{16}}$
$c_{67}$	$-\frac{94162698713311\,b_{2}\,{\left( \frac{\tan \theta _0}{2}\right) }^{554855/32768}}{47829450699604754432\,{\tan \theta _0}^{17}}$	$c_{68}$	$\frac{388717606998977\,b_{2}\,{\left( \frac{\tan \theta _0}{2}\right) }^{587623/32768}}{1519623251576745885696\,{\tan \theta _0}^{18}}$
$c_{69}$	$-\frac{1057140962472757\,b_{2}\,{\left( \frac{\tan \theta _0}{2}\right) }^{620391/32768}}{52154529959914841833472\,{\tan \theta _0}^{19}}$	$c_{70}$	$\frac{2775672680954111\,b_{2}\,{\left( \frac{\tan \theta _0}{2}\right) }^{653159/32768}}{3707279049981556007370752\,{\tan \theta _0}^{20}}$

**Table 3:** Asymptotic expansion constants for $\Sigma _2^\infty$

Constant	Value	Constant	Value
$d_{1,1}$	$\frac{\tan ^3\theta _0}{2(\frac{1}{\alpha } - 2)}$	$d_{1,2}$	$- \frac{3\tan ^5\theta _0}{8(\frac{1}{\alpha } - 4)}$
$d_{1,3}$	$\frac{5\tan ^7\theta _0}{16(\frac{1}{\alpha } - 6)}$	$d_{1,4}$	$\frac{b_1\tan ^5\theta _0}{8(\frac{1}{\alpha } - 4)}$
$d_{1,5}$	$- \frac{b_1\tan ^7\theta _0}{8(\frac{1}{\alpha } - 6)}$	$d_{1,6}$	$- \frac{5b_1 \tan ^9\theta _0}{32(\frac{1}{\alpha } - 8)}$
$d_{2,1}$	$\frac{\tan ^3\theta _0}{2(2(\delta + \tan \theta _0)^2 -\frac{ (\delta + \tan \theta _0)^2}{\alpha })}$	$d_{2,2}$	$- \frac{3\tan ^5\theta _0}{8(4(\delta + \tan \theta _0)^4 - \frac{(\delta + \tan \theta _0)^4}{\alpha })}$
$d_{2,3}$	$\frac{5\tan ^7\theta _0}{16(6(\delta + \tan \theta _0)^6 - \frac{(\delta +\tan \theta _0 )^6}{\alpha })}$	$d_{2,4}$	$\frac{b_1\tan ^5\theta _0}{8(4(\delta + \tan \theta _0)^4 - \frac{(\delta + \tan \theta _0)^4}{\alpha })}$
$d_{2,5}$	$- \frac{b_1\tan ^7\theta _0}{8(6(\delta + \tan \theta _0)^6 - \frac{(\delta + \tan \theta _0)^6}{\alpha })}$	$d_{2,6}$	$- \frac{5b_1\tan ^9\theta _0}{32(8(\delta + \tan \theta _0)^8 - \frac{(\delta + \tan \theta _0)^8}{\alpha })}$
$d_{2,7}$	$\frac{3916342628302019\,b_{2}\,{\left( \delta +\tan \theta _0\right) }^{\frac{391015}{32768}}}{2048\,\left( 3306312129576960\,{\tan \theta _0}^{12}+\frac{277076930199552\,{\tan \theta _0}^{12}}{\alpha }\right) }$	$d_{2,8}$	$-\frac{4112068129940143\,b_{2}\,{\left( \delta +\tan \theta _0\right) }^{\frac{423783}{32768}}}{131072\,\left( 895847264944128\,{\tan \theta _0}^{13}+\frac{69269232549888\,{\tan \theta _0}^{13}}{\alpha }\right) }$
$d_{2,9}$	$-\frac{3363754402794477\,b_{2}\,{\left( \delta +\tan \theta _0\right) }^{\frac{456551}{32768}}}{4096\,\left( 13511630964916224\,{\tan \theta _0}^{14}+\frac{969769255698432\,{\tan \theta _0}^{14}}{\alpha }\right) }$	$d_{2,10}$	$-\frac{5083615087327005\,b_{2}\,{\left( \delta +\tan \theta _0\right) }^{\frac{522087}{32768}}}{2048\,\left( 247218711621009408\,{\tan \theta _0}^{16}+\frac{15516308091174912\,{\tan \theta _0}^{16}}{\alpha }\right) }$
$d_{2,11}$	$-\frac{2255234053275215\,b_{2}\,{\left( \delta +\tan \theta _0\right) }^{\frac{653159}{32768}}}{4294967296\,\left( 701324136022016\,{\tan \theta _0}^{20}+\frac{35184372088832\,{\tan \theta _0}^{20}}{\alpha }\right) }$	$d_{2,12}$	$-\frac{737389043420589\,b_{2}\,{\left( \delta +\tan \theta _0\right) }^{\frac{128871}{32768}}}{32768\,\left( 540524150784\,{\tan \theta _0}^4+\frac{137438953472\,{\tan \theta _0}^4}{\alpha }\right) }$
$d_{2,13}$	$\frac{7868723041398407\,b_{2}\,{\left( \delta +\tan \theta _0\right) }^{\frac{63335}{32768}}}{33554432\,\left( 16602890240\,{\tan \theta _0}^2+\frac{8589934592\,{\tan \theta _0}^2}{\alpha }\right) }$	$d_{2,14}$	$\frac{2682583518496071\,b_{2}\,{\left( \delta +\tan \theta _0\right) }^{\frac{161639}{32768}}}{262144\,\left( 338981552128\,{\tan \theta _0}^5+\frac{68719476736\,{\tan \theta _0}^5}{\alpha }\right) }$
$d_{2,15}$	$-\frac{187\,\sqrt{2}\,{\left( \delta +\tan \theta _0\right) }}{\frac{256}{\alpha }+256}$	$d_{2,16}$	$-\frac{15\,\sqrt{2}\,{\left( \delta +\tan \theta _0\right) }^{4}}{256\,{\tan \theta _0}^3+\frac{64\,{\tan \theta _0}^3}{\alpha }}$
$d_{2,17}$	$\frac{43\,\sqrt{2}\,{\left( \delta +\tan \theta _0\right) }^{3}}{384\,{\tan \theta _0}^2+\frac{128\,{\tan \theta _0}^2}{\alpha }}$	$d_{2,18}$	$\frac{13\,\sqrt{2}\,{\left( \delta +\tan \theta _0\right) }^{5}}{1280\,{\tan \theta _0}^4+\frac{256\,{\tan \theta _0}^4}{\alpha }}$
$d_{2,19}$	$\frac{3171422887418271\,b_{2}\,{\left( \delta +\tan \theta _0\right) }^{\frac{620391}{32768}}}{134217728\,\left( 1165744586883072\,{\tan \theta _0}^{19}+\frac{61572651155456\,{\tan \theta _0}^{19}}{\alpha }\right) }$	$d_{2,20}$	$\frac{3198993443274073\,b_{2}\,{\left( \delta +\tan \theta _0\right) }^{\frac{227175}{32768}}}{32768\,\left( 7503622963200\,{\tan \theta _0}^7+\frac{1082331758592\,{\tan \theta _0}^7}{\alpha }\right) }$
$d_{2,21}$	$\frac{7704243768453287\,b_{2}\,{\left( \delta +\tan \theta _0\right) }^{\frac{96103}{32768}}}{16777216\,\left( 151156948992\,{\tan \theta _0}^3+\frac{51539607552\,{\tan \theta _0}^3}{\alpha }\right) }$	$d_{2,22}$	$\frac{5\,\sqrt{2}\,{\left( \delta +\tan \theta _0\right) }^{2}}{128\,\tan \theta _0+\frac{64\,\tan \theta _0}{\alpha }}$
$d_{2,23}$	$\frac{4515412343364391\,b_{2}\,{\left( \delta +\tan \theta _0\right) }^{\frac{30567}{32768}}}{34359738368\,\left( 978144\,\tan \theta _0+\frac{1048576\,\tan \theta _0}{\alpha }\right) }$	$d_{2,24}$	$-\frac{7116713214246575\,b_{2}\,{\left( \delta +\tan \theta _0\right) }^{\frac{194407}{32768}}}{32768\,\left( 14677237039104\,{\tan \theta _0}^6+\frac{2473901162496\,{\tan \theta _0}^6}{\alpha }\right) }$
$d_{2,25}$	$\frac{813773447008073\,b_{2}\,{\left( \delta +\tan \theta _0\right) }^{\frac{292711}{32768}}}{2048\,\left( 309385167372288\,{\tan \theta _0}^9+\frac{34634616274944\,{\tan \theta _0}^9}{\alpha }\right) }$	$d_{2,26}$	$\frac{5899216819711551\,b_{2}\,{\left( \delta +\tan \theta _0\right) }^{\frac{325479}{32768}}}{8192\,\left( 344019783647232\,{\tan \theta _0}^{10}+\frac{34634616274944\,{\tan \theta _0}^{10}}{\alpha }\right) }$
$d_{2,27}$	$-\frac{5278606494654121\,b_{2}\,{\left( \delta +\tan \theta _0\right) }^{\frac{358247}{32768}}}{8192\,\left( 378654399922176\,{\tan \theta _0}^{11}+\frac{34634616274944\,{\tan \theta _0}^{11}}{\alpha }\right) }$	$d_{2,28}$	$\frac{1772224420073771\,b_{2}\,{\left( \delta +\tan \theta _0\right) }^{\frac{489319}{32768}}}{65536\,\left( 804522234478592\,{\tan \theta _0}^{15}+\frac{53876069761024\,{\tan \theta _0}^{15}}{\alpha }\right) }$
$d_{2,29}$	$\frac{470813493566555\,b_{2}\,{\left( \delta +\tan \theta _0\right) }^{\frac{554855}{32768}}}{32768\,\left( 7298194992005120\,{\tan \theta _0}^{17}+\frac{431008558088192\,{\tan \theta _0}^{17}}{\alpha }\right) }$	$d_{2,30}$	$-\frac{1390888037066579\,b_{2}\,{\left( \delta +\tan \theta _0\right) }^{\frac{259943}{32768}}}{1024\,\left( 157000314912768\,{\tan \theta _0}^8+\frac{19791209299968\,{\tan \theta _0}^8}{\alpha }\right) }$
$d_{2,31}$	$-\frac{388717606998977\,b_{2}\,{\left( \delta +\tan \theta _0\right) }^{\frac{587623}{32768}}}{32768\,\left( 46375221300559872\,{\tan \theta _0}^{18}+\frac{2586051348529152\,{\tan \theta _0}^{18}}{\alpha }\right) }$	$d_{2,32}$	$\frac{187\,\sqrt{2}\,b_{2}\,{\left( \delta +\tan \theta _0\right) }^{-\frac{2201}{32768}}}{\frac{256}{\alpha }-\frac{2201}{128}}$
$d_{3,1}$	$-\frac{3198993443274073\,b_{2}\,{\left( \frac{\tan \theta _0}{2}\right) }^{\frac{227175}{32768}}}{32768\,\left( 7503622963200\,{\tan \theta _0}^7+\frac{1082331758592\,{\tan \theta _0}^7}{\alpha }\right) }$	$d_{3,2}$	$-\frac{3171422887418271\,b_{2}\,{\left( \frac{\tan \theta _0}{2}\right) }^{\frac{620391}{32768}}}{134217728\,\left( 1165744586883072\,{\tan \theta _0}^{19}+\frac{61572651155456\,{\tan \theta _0}^{19}}{\alpha }\right) }$
$d_{3,3}$	$-\frac{7704243768453287\,b_{2}\,{\left( \frac{\tan \theta _0}{2}\right) }^{\frac{96103}{32768}}}{16777216\,\left( 151156948992\,{\tan \theta _0}^3+\frac{51539607552\,{\tan \theta _0}^3}{\alpha }\right) }$	$d_{3,4}$	$-\frac{5\,\sqrt{2}\,{\left( \frac{\tan \theta _0}{2}\right) }^{2}}{128\,\tan \theta _0+\frac{64\,\tan \theta _0}{\alpha }}$
$d_{3,5}$	$-\frac{4515412343364391\,b_{2}\,{\left( \frac{\tan \theta _0}{2}\right) }^{\frac{30567}{32768}}}{34359738368\,\left( 978144\,\tan \theta _0+\frac{1048576\,\tan \theta _0}{\alpha }\right) }$	$d_{3,6}$	$\frac{7116713214246575\,b_{2}\,{\left( \frac{\tan \theta _0}{2}\right) }^{\frac{194407}{32768}}}{32768\,\left( 14677237039104\,{\tan \theta _0}^6+\frac{2473901162496\,{\tan \theta _0}^6}{\alpha }\right) }$
$d_{3,7}$	$-\frac{813773447008073\,b_{2}\,{\left( \frac{\tan \theta _0}{2}\right) }^{\frac{1}{\alpha }+\frac{292711}{32768}}}{2048\,\left( 309385167372288\,{\tan \theta _0}^9+\frac{34634616274944\,{\tan \theta _0}^9}{\alpha }\right) }$	$d_{3,8}$	$-\frac{5899216819711551\,b_{2}\,{\left( \frac{\tan \theta _0}{2}\right) }^{\frac{325479}{32768}}}{8192\,\left( 344019783647232\,{\tan \theta _0}^{10}+\frac{34634616274944\,{\tan \theta _0}^{10}}{\alpha }\right) }$
$d_{3,9}$	$\frac{5278606494654121\,b_{2}\,{\left( \frac{\tan \theta _0}{2}\right) }^{\frac{358247}{32768}}}{8192\,\left( 378654399922176\,{\tan \theta _0}^{11}+\frac{34634616274944\,{\tan \theta _0}^{11}}{\alpha }\right) }$	$d_{3,10}$	$-\frac{1772224420073771\,b_{2}\,{\left( \frac{\tan \theta _0}{2}\right) }^{\frac{489319}{32768}}}{65536\,\left( 804522234478592\,{\tan \theta _0}^{15}+\frac{53876069761024\,{\tan \theta _0}^{15}}{\alpha }\right) }$
$d_{3,11}$	$-\frac{470813493566555\,b_{2}\,{\left( \frac{\tan \theta _0}{2}\right) }^{\frac{554855}{32768}}}{32768\,\left( 7298194992005120\,{\tan \theta _0}^{17}+\frac{431008558088192\,{\tan \theta _0}^{17}}{\alpha }\right) }$	$d_{3,12}$	$\frac{1390888037066579\,b_{2}\,{\left( \frac{\tan \theta _0}{2}\right) }^{\frac{259943}{32768}}}{1024\,\left( 157000314912768\,{\tan \theta _0}^8+\frac{19791209299968\,{\tan \theta _0}^8}{\alpha }\right) }$
$d_{3,13}$	$\frac{388717606998977\,b_{2}\,{\left( \frac{\tan \theta _0}{2}\right) }^{\frac{587623}{32768}}}{32768\,\left( 46375221300559872\,{\tan \theta _0}^{18}+\frac{2586051348529152\,{\tan \theta _0}^{18}}{\alpha }\right) }$	$d_{3,14}$	$\frac{4112068129940143\,b_{2}\,{\left( \frac{\tan \theta _0}{2}\right) }^{\frac{423783}{32768}}}{131072\,\left( 895847264944128\,{\tan \theta _0}^{13}+\frac{69269232549888\,{\tan \theta _0}^{13}}{\alpha }\right) }$
$d_{3,15}$	$\frac{3363754402794477\,b_{2}\,{\left( \frac{\tan \theta _0}{2}\right) }^{\frac{456551}{32768}}}{4096\,\left( 13511630964916224\,{\tan \theta _0}^{14}+\frac{969769255698432\,{\tan \theta _0}^{14}}{\alpha }\right) }$	$d_{3,16}$	$\frac{5083615087327005\,b_{2}\,{\left( \frac{\tan \theta _0}{2}\right) }^{\frac{522087}{32768}}}{2048\,\left( 247218711621009408\,{\tan \theta _0}^{16}+\frac{15516308091174912\,{\tan \theta _0}^{16}}{\alpha }\right) }$
$d_{3,17}$	$\frac{2255234053275215\,b_{2}\,{\left( \frac{\tan \theta _0}{2}\right) }^{\frac{653159}{32768}}}{4294967296\,\left( 701324136022016\,{\tan \theta _0}^{20}+\frac{35184372088832\,{\tan \theta _0}^{20}}{\alpha }\right) }$	$d_{3,18}$	$-\frac{3916342628302019\,b_{2}\,{\left( \frac{\tan \theta _0}{2}\right) }^{\frac{391015}{32768}}}{2048\,\left( 3306312129576960\,{\tan \theta _0}^{12}+\frac{277076930199552\,{\tan \theta _0}^{12}}{\alpha }\right) }$
$d_{3,19}$	$\frac{737389043420589\,b_{2}\,{\left( \frac{\tan \theta _0}{2}\right) }^{\frac{128871}{32768}}}{32768\,\left( 540524150784\,{\tan \theta _0}^4+\frac{137438953472\,{\tan \theta _0}^4}{\alpha }\right) }$	$d_{3,20}$	$-\frac{7868723041398407\,b_{2}\,{\left( \frac{\tan \theta _0}{2}\right) }^{\frac{63335}{32768}}}{33554432\,\left( 16602890240\,{\tan \theta _0}^2+\frac{8589934592\,{\tan \theta _0}^2}{\alpha }\right) }$
$d_{3,21}$	$-\frac{2682583518496071\,b_{2}\,{\left( \frac{\tan \theta _0}{2}\right) }^{\frac{161639}{32768}}}{262144\,\left( 338981552128\,{\tan \theta _0}^5+\frac{68719476736\,{\tan \theta _0}^5}{\alpha }\right) }$	$d_{3,22}$	$\frac{187\,\sqrt{2}\,{\left( \frac{\tan \theta _0}{2}\right) }}{\frac{256}{\alpha }+256}$
$d_{3,23}$	$\frac{15\,\sqrt{2}\,{\left( \frac{\tan \theta _0}{2}\right) }^{4}}{256\,{\tan \theta _0}^3+\frac{64\,{\tan \theta _0}^3}{\alpha }}$	$d_{3,24}$	$-\frac{43\,\sqrt{2}\,{\left( \frac{\tan \theta _0}{2}\right) }^{3}}{384\,{\tan \theta _0}^2+\frac{128\,{\tan \theta _0}^2}{\alpha }}$
$d_{3,25}$	$-\frac{13\,\sqrt{2}\,{\left( \frac{\tan \theta _0}{2}\right) }^{5}}{1280\,{\tan \theta _0}^4+\frac{256\,{\tan \theta _0}^4}{\alpha }}$	$d_{3,26}$	$-\frac{187\,\sqrt{2}\,b_{2}\,{\left( \frac{\tan \theta _0}{2}\right) }^{-\frac{2201}{32768}}}{\frac{256}{\alpha }-\frac{2201}{128}}$
$d_{3,27}$	$- \frac{\frac{\tan \theta _0}{2}}{\frac{1}{\alpha } + 1}$	$d_{3,28}$	$\frac{\frac{\tan \theta _0}{2}^{3}}{2(3\tan ^2\theta _0 + \frac{\tan ^2\theta _0}{\alpha })}$
$d_{3,29}$	$- \frac{3\frac{\tan \theta _0}{2}^{ 5}}{8(5\tan ^4\theta _0 + \frac{\tan ^4\theta _0}{\alpha })}$	$d_{3,30}$	$\frac{5\frac{\tan \theta _0}{2}^{7}}{16(7\tan ^6\theta _0 + \frac{\tan ^6\theta _0}{\alpha })}$
$d_{3,31}$	$\frac{b_3\frac{\tan \theta _0}{2}^{ 4}}{8(4\tan ^4\theta _0 + \frac{\tan ^4\theta _0}{\alpha })}$	$d_{3,32}$	$- \frac{b_3\frac{\tan \theta _0}{2}^{6}}{8(6\tan ^6\theta _0 + \frac{\tan ^6\theta _0}{\alpha })}$
$d_{3,33}$	$- \frac{5b_3\frac{\tan \theta _0}{2}^{8}}{32(8\tan ^8\theta _0 + \frac{\tan ^8\theta _0}{\alpha })}$		
$d_{4,1}$	$\frac{3\chi _{\text {hot}}^{ 5}}{8(5\tan ^4\theta _0 + \frac{\tan ^4\theta _0}{\alpha })}$	$d_{4,2}$	$- \frac{\chi _{\text {hot}}^{ 3}}{2(3\tan ^2\theta _0 + \frac{\tan ^2\theta _0}{\alpha })}$
$d_{4,3}$	$- \frac{5\chi _{\text {hot}}^{7}}{16(7\tan ^6\theta _0 + \frac{\tan ^6\theta _0}{\alpha })}$	$d_{4,4}$	$\frac{\chi _{\text {hot}}}{\frac{1}{\alpha } + 1}$
$d_{4,5}$	$- \frac{b_3\chi _{\text {hot}}^{4}}{8(4\tan ^4\theta _0 + \frac{\tan ^4\theta _0}{\alpha })}$	$d_{4,6}$	$\frac{b_3 \chi _{\text {hot}}^{6}}{8(6\tan ^6\theta _0 + \frac{\tan ^6\theta _0}{\alpha })}$
$d_{4,7}$	$\frac{5 b_3\chi _{\text {hot}}^{ 8}}{32(8\tan ^8\theta _0 + \frac{\tan ^8\theta _0}{\alpha })}$		

We have all the needed approximations to calculate the stress resultant analytically. We start of with the cellulose contribution independent of the biomechanical hotspots such that,

F78 $$\begin{aligned} \Sigma _1^\infty =&\int ^1_\epsilon (L_c-1)\sin \theta \ \text {d}y \nonumber \\&=\int ^1_{\tan \theta _0+\delta }\left( b_1\left( 1+\frac{\tan ^2\theta _0}{2\hat{y}^2}\right) -1\right) \nonumber \\&\quad \times \left( \frac{\tan \theta _0}{y}-\frac{1}{2}\frac{\tan ^3\theta _0}{y^3}+\frac{3}{8}\frac{\tan ^5\theta _0}{y^5} -\frac{5}{16}\frac{\tan ^7\theta _0}{y^7}\right) \ \text {d}y \nonumber \\&+\int ^{\tan \theta _0+\delta }_{\frac{\tan \theta _0}{2}} \left( \frac{b_2}{y^{\frac{34969}{32768}}} \left( 1-H(y)+\frac{H(y)^2}{2}\right) -1\right) \nonumber \\&\quad \times \left( \frac{1}{\sqrt{2}}-\frac{y-\tan \theta _0}{2\sqrt{2}\tan \theta _0} +\frac{(y-\tan \theta )^2}{8\sqrt{2}\tan ^2\theta _0}\right. \nonumber \\&\left. +\frac{(y-\tan \theta )^3}{16\sqrt{2}\tan ^3\theta _0}- \frac{13 (y - \tan \theta _0)^4}{128 \sqrt{2}\tan \theta _0^4}\right) \ \text {d}y \nonumber \\&+\int ^{\frac{\tan \theta _0}{2}}_\epsilon \left( \frac{b_3}{y}\left( 1+\frac{y^2}{2\tan ^2\theta _0}\right) -1\right) \nonumber \\&\quad \times \left( 1-\frac{y^2}{2\tan ^2\theta _0}+\frac{3y^4}{8\tan ^4\theta _0}-\frac{5y^6}{16\tan ^6\theta _0}\right) \ \text {d}y. \end{aligned}$$

which we find to be

F79 $$\begin{aligned} \Sigma _1^\infty = \sum _i c_{i}, \end{aligned}$$

where all the $c_i$ values can be found in Table 2.

The second term is the contribution from the cellulose fibres when crosslinked by the biomechanical hotspots which we calculate to be,

F80 $$\begin{aligned} \Sigma _2^\infty =&\int ^1_\epsilon a_1 n_{\text {hot}} (L_c-1)\sin \theta \ \text {d}y \nonumber \\&=\int ^1_{\tan \theta _0+\delta }a_1y^{\frac{1}{\alpha }}\left( b_1\left( 1+\frac{\tan ^2\theta _0}{2\hat{y}^2}\right) -1\right) \nonumber \\&\quad \times \left( \frac{\tan \theta _0}{y} -\frac{1}{2}\frac{\tan ^3\theta _0}{y^3}+\frac{3}{8}\frac{\tan ^5\theta _0}{y^5} -\frac{5}{16}\frac{\tan ^7\theta _0}{y^7}\right) \ \text {d}y\nonumber \\&\quad +\int ^{\tan \theta _0+\delta }_{\frac{\tan \theta _0}{2}}a_1y^{\frac{1}{\alpha }} \left( \frac{b_2}{y^{\frac{34969}{32768}}} (1-H(y)+\frac{H(y)^2}{2})-1\right) \nonumber \\&\quad \times \left( \frac{1}{\sqrt{2}}-\frac{y-\tan \theta _0}{2\sqrt{2}\tan \theta _0}+\frac{(y-\tan \theta )^2}{8\sqrt{2}\tan ^2\theta _0}\right. \nonumber \\&\quad \left. +\frac{(y-\tan \theta )^3}{16\sqrt{2}\tan ^3\theta _0} - \frac{13 (y - \tan \theta _0)^4}{128 \sqrt{2}\tan \theta _0^4}\right) \ \text {d}y \nonumber \\&\quad +\int ^{\frac{\tan \theta _0}{2}}_{\chi _{\text {hot}}} a_1 y^{\frac{1}{\alpha }} \left( \frac{b_3}{y}\left( 1+\frac{y^2}{2\tan ^2\theta _0}\right) -1\right) \nonumber \\&\quad \times \left( 1-\frac{y^2}{2\tan ^2\theta _0}+\frac{3y^4}{8\tan ^4\theta _0}-\frac{5y^6}{16\tan ^6\theta _0}\right) \ \text {d}y \nonumber \\&+\int ^{\chi _{\text {hot}}}_\epsilon a_1 \exp \left( -\frac{1}{\alpha }\frac{y^2}{2\beta _{\text {hot}}^2}e^{\frac{\beta _{\text {hot}}^2}{y^2}} \right) \nonumber \\&\quad \times \left( \frac{b_3}{y} \left( 1+\frac{y^2}{2\tan ^2\theta _0}\right) -1\right) \nonumber \\&\quad \times \left( 1-\frac{y^2}{2\tan ^2\theta _0} +\frac{3y^4}{8\tan ^4\theta _0}-\frac{5y^6}{16\tan ^6\theta _0}\right) \ \text {d}y \end{aligned}$$

which becomes

F81 $$\begin{aligned} \Sigma _{2}^\infty =&a_1\left( \left( \sum _i d_{1,i}(\alpha )\right) + \tan _0\left( b_1-1\right) \alpha \right. \nonumber \\&+ \left( \sum _i d_{2,i}(\alpha )\right) (\delta + \tan \theta _0)^{\frac{1}{\alpha }}\nonumber \\&\left. + \tan \theta _0\left( 1-b_1\right) \alpha (\delta + \tan \theta _0)^{\frac{1}{\alpha }}\right. \nonumber \\&\left. + \left( \sum _i d_{3,i}(\alpha )\right) \left( \frac{\tan \theta _0}{2}\right) ^{\frac{1}{\alpha }}\right. \nonumber \\&\left. + b_3 \alpha \left( \frac{\tan \theta _0}{2}\right) ^{\frac{1}{\alpha }} +\left( \sum _i d_{4,i}(\alpha )\right) \right. \nonumber \\&\left. \quad (\chi _{\text {hot}})^{\frac{1}{\alpha }} -b_3 \alpha (\chi _{\text {hot}})^{\frac{1}{\alpha }}\right) \end{aligned}$$

where the values of the coefficients $d_{1,i},\ldots ,$,$d_{2,i},\ldots ,$, $d_{3,i},\ldots ,$, and $d_{4,i},\ldots ,$ can be found in Table 3.

The third term is the hemicellulose contribution and is found to be,

F82 $$\begin{aligned} \Sigma _3^\infty =&\int ^1_\epsilon a_2 n n_{\text {hot}} \sigma _H \ \text {d}y=\int ^1_{\chi } a_2 \breve{\alpha }y^{\frac{1}{\alpha }}y^{\frac{\breve{k}_0}{\alpha }} \left( \frac{1}{y}-y^{\frac{\omega }{\alpha }}\right) \ \text {d}y \nonumber \\&+\int ^{\chi }_{\chi _{\text {hot}}} +a_2\breve{\alpha }y^{\frac{1}{\alpha }} \exp \left( -\frac{\breve{k}_0}{\alpha }\frac{y^2}{2\breve{\alpha }^2\gamma ^2}e^{\breve{\alpha }^2 \frac{\gamma ^2}{y^2}} \right) \left( \frac{1}{y}-y^{\frac{\omega }{\alpha }}\right) \ \text {d}y \nonumber \\&+\int ^{\chi _{\text {hot}}}_\epsilon a_2\breve{\alpha }\exp \left( -\frac{1}{\alpha } \frac{y^2}{2\beta _{\text {hot}}^2}e^{\frac{\beta _{\text {hot}}^2}{y^2}} \right) \nonumber \\&\quad \times \exp \left( -\frac{\breve{k}_0}{\alpha }\frac{y^2}{2\breve{\alpha }^2\gamma ^2}e^{\frac{\breve{\alpha }^2\gamma ^2}{y^2}} \right) \left( \frac{1}{y}-y^{\frac{\omega }{\alpha }}\right) \ \text {d}y \end{aligned}$$

where upon integration

F83 $$\begin{aligned} \Sigma _3^\infty =a_2\breve{\alpha }&\left( \frac{\alpha }{\breve{k}_0+1}\left( 1{-}\chi ^{\frac{\breve{k}_0{+}1}{\alpha }}\right) \right. \nonumber \\&\left. -\frac{1}{\frac{1}{\alpha }\left( \breve{k}_0{+}\omega {+}1\right) {+}1} \left( 1-\chi ^{\frac{\breve{k}_0+\omega +1}{\alpha }+1}\right) \right) \end{aligned}$$

Note that for all three contributions to the stress resultant, $\Sigma _1^\infty$, $\Sigma _2^\infty$, and $\Sigma _3^\infty$ for some critical values of $\alpha$ some of their terms are logarithmic upon integration which we choose to ignore.

## References

[1] I. Ahuja, R.C. de Vos, A.M. Bones, R.D. Hall, Plant molecular stress responses face climate change. Trends Plant Sci. **15**(12), 664–674 (2010) 20846898 10.1016/j.tplants.2010.08.002

[2] J. Flood, The importance of plant health to food security. Food Secur. **2**(3), 215–231 (2010)

[3] D.J. Cosgrove, Growth of the plant cell wall. Nat. Rev. Mol. Cell Biol. **6**(11), 850–861 (2005) 16261190 10.1038/nrm1746

[4] J.A. Lockhart, An analysis of irreversible plant cell elongation. J. Theor. Biol. **8**(2), 264–275 (1965) 5876240 10.1016/0022-5193(65)90077-9

[5] H. Höfte, A. Peaucelle, S. Braybrook, Cell wall mechanics and growth control in plants: the role of pectins revisited. Front. Plant Sci. **3**, 121 (2012). 10.3389/fpls.2012.00121 22685449 10.3389/fpls.2012.00121 PMC3368173

[6] E.T. Smithers, J. Luo, R.J. Dyson, Mathematical principles and models of plant growth mechanics: from cell wall dynamics to tissue morphogenesis. J. Exp. Bot. **70**(14), 3587–3600 (2019) 31128070 10.1093/jxb/erz253

[7] A. Geitmann, J.K. Ortega, Mechanics and modeling of plant cell growth. Trends Plant Sci. **14**(9), 467–478 (2009) 19717328 10.1016/j.tplants.2009.07.006

[8] O. Ali, V. Mirabet, C. Godin, J. Traas, Physical models of plant development. Annu. Rev. Cell Dev. Biol. **30**, 59–78 (2014) 25000996 10.1146/annurev-cellbio-101512-122410

[9] D.J. Cosgrove, Re-constructing our models of cellulose and primary cell wall assembly. Curr. Opin. Plant Biol. **22**, 122–131 (2014) 25460077 10.1016/j.pbi.2014.11.001 PMC4293254

[10] D.J. Cosgrove, Catalysts of plant cell wall loosening. *F1000Research* **5** (2016). 10.12688/f1000research.7180.1 10.12688/f1000research.7180.1 PMC4755413 26918182

[11] D.J. Cosgrove, Building an extensible cell wall. Technical report, Northwestern Univ., Evanston, IL (United States); Energy Frontier Research (2022)

[12] T. Zhang, H. Tang, D. Vavylonis, D.J. Cosgrove, Disentangling loosening from softening: insights into primary cell wall structure. Plant J. **100**(6), 1101–1117 (2019) 31469935 10.1111/tpj.14519

[13] H.V. Scheller, P. Ulvskov, Hemicelluloses. Annu. Rev. Plant Biol. **61**, 263–289 (2010) 20192742 10.1146/annurev-arplant-042809-112315

[14] Y.B. Park, D.J. Cosgrove, Xyloglucan and its interactions with other components of the growing cell wall. Plant Cell Physiol. **56**(2), 180–194 (2015) 25613914 10.1093/pcp/pcu204

[15] M.C. Jarvis, Plant cell walls: supramolecular assembly, signalling and stress. Struct. Chem. **20**(2), 245–253 (2009)

[16] T. Zhang, Y. Zheng, D.J. Cosgrove, Spatial organization of cellulose microfibrils and matrix polysaccharides in primary plant cell walls as imaged by multichannel atomic force microscopy. Plant J. **85**(2), 179–192 (2016) 26676644 10.1111/tpj.13102

[17] Y. Zhang, J. Yu, X. Wang, D.M. Durachko, S. Zhang, D.J. Cosgrove, Molecular insights into the complex mechanics of plant epidermal cell walls. Science **372**(6543), 706–711 (2021) 33986175 10.1126/science.abf2824

[18] C.T. Anderson, J.J. Kieber, Dynamic construction, perception, and remodeling of plant cell walls. Annu. Rev. Plant Biol. **71**, 39–69 (2020) 32084323 10.1146/annurev-arplant-081519-035846

[19] C.T. Anderson, A. Carroll, L. Akhmetova, C. Somerville, Real-time imaging of cellulose reorientation during cell wall expansion in arabidopsis roots. Plant Physiol. **152**(2), 787–796 (2010) 19965966 10.1104/pp.109.150128 PMC2815888

[20] I. Burgert, P. Fratzl, Plants control the properties and actuation of their organs through the orientation of cellulose fibrils in their cell walls. Integr. Comp. Biol. **49**(1), 69–79 (2009) 21669847 10.1093/icb/icp026

[21] A. Geitmann, Mechanical modeling and structural analysis of the primary plant cell wall. Curr. Opin. Plant Biol. **13**(6), 693–699 (2010) 20971032 10.1016/j.pbi.2010.09.017

[22] K. Kafle, X. Xi, C.M. Lee, B.R. Tittmann, D.J. Cosgrove, Y.B. Park, S.H. Kim, Cellulose microfibril orientation in onion (allium cepa l.) epidermis studied by atomic force microscopy (afm) and vibrational sum frequency generation (sfg) spectroscopy. Cellulose **21**(2), 1075–1086 (2014)

[23] D. Chen, L.D. Melton, D.J. McGillivray, T.M. Ryan, P.J. Harris, Changes in the orientations of cellulose microfibrils during the development of collenchyma cell walls of celery (apium graveolens l.). Planta **250**, 1819–1832 (2019) 31463558 10.1007/s00425-019-03262-8

[24] D.S. Thompson, How do cell walls regulate plant growth? J. Exp. Bot. **56**(419), 2275–2285 (2005) 16061505 10.1093/jxb/eri247

[25] H. Yi, V.M. Puri, Architecture-based multiscale computational modeling of plant cell wall mechanics to examine the hydrogen-bonding hypothesis of cell wall network structure model. Plant Physiol. **160**(3), 1281–1292 (2012) 22926320 10.1104/pp.112.201228 PMC3490585

[26] D.J. Cosgrove, Plant cell wall extensibility: connecting plant cell growth with cell wall structure, mechanics, and the action of wall-modifying enzymes. J. Exp. Bot. **67**(2), 463–476 (2015) 26608646 10.1093/jxb/erv511

[27] A. Nili, H. Yi, V.H. Crespi, V.M. Puri, Examination of biological hotspot hypothesis of primary cell wall using a computational cell wall network model. Cellulose **22**(2), 1027–1038 (2015)

[28] S. Yuan, Y. Wu, D.J. Cosgrove, A fungal endoglucanase with plant cell wall extension activity. Plant Physiol. **127**(1), 324–333 (2001) 11553760 10.1104/pp.127.1.324 PMC117988

[29] J. Sampedro, D.J. Cosgrove, The expansin superfamily. Genome Biol. **6**(12), 242 (2005) 16356276 10.1186/gb-2005-6-12-242 PMC1414085

[30] D.J. Cosgrove, Loosening of plant cell walls by expansins. Nature **407**(6802), 321 (2000) 11014181 10.1038/35030000

[31] G. Arsuffi, S.A. Braybrook, Acid growth: an ongoing trip. J. Exp. Bot. **69**(2), 137–146 (2017) 10.1093/jxb/erx390 29211894

[32] A. Creff, L. Brocard, G. Ingram, A mechanically sensitive cell layer regulates the physical properties of the arabidopsis seed coat. Nat. Commun. **6**, 6382 (2015) 25702924 10.1038/ncomms7382

[33] P. Derbyshire, K. Findlay, M.C. McCann, K. Roberts, Cell elongation in arabidopsis hypocotyls involves dynamic changes in cell wall thickness. J. Exp. Bot. **58**(8), 2079–2089 (2007) 17470442 10.1093/jxb/erm074

[34] T.I. Baskin, O.E. Jensen, On the role of stress anisotropy in the growth of stems. J. Exp. Bot. **64**(15), 4697–4707 (2013) 23913952 10.1093/jxb/ert176

[35] R. Dyson, L. Band, O. Jensen, A model of crosslink kinetics in the expanding plant cell wall: yield stress and enzyme action. J. Theor. Biol. **307**, 125–136 (2012) 22584249 10.1016/j.jtbi.2012.04.035 PMC3414840

[36] J.E.F. Green, A. Friedman, The extensional flow of a thin sheet of incompressible, transversely isotropic fluid. Eur. J. Appl. Math. **19**(3), 225–257 (2008)

[37] P. Sotiriou, E. Giannoutsou, E. Panteris, B. Galatis, P. Apostolakos, Local differentiation of cell wall matrix polysaccharides in sinuous pavement cells: its possible involvement in the flexibility of cell shape. Plant Biol. **20**(2), 223–237 (2018) 29247575 10.1111/plb.12681

[38] A.J. Bidhendi, A. Geitmann, Relating the mechanics of the primary plant cell wall to morphogenesis. J. Exp. Bot. **67**(2), 449–461 (2015) 26689854 10.1093/jxb/erv535

[39] M. Dembo, D. Torney, K. Saxman, D. Hammer, The reaction-limited kinetics of membrane-to-surface adhesion and detachment. Proc. R. Soc. Lond. Ser. B **234**(1274), 55–83 (1988) 2901109 10.1098/rspb.1988.0038

[40] N. Li, S. Lü, Y. Zhang, M. Long, Mechanokinetics of receptor-ligand interactions in cell adhesion. Acta. Mech. Sin. **31**(2), 248–258 (2015)

[41] R.D. Groot, A. Bot, W.G. Agterof, Molecular theory of the yield behavior of a polymer gel: application to gelatin. J. Chem. Phys. **104**(22), 9220–9233 (1996)

[42] G.I. Bell, M. Dembo, P. Bongrand, Cell adhesion. competition between nonspecific repulsion and specific bonding. Biophys. J . **45**(6), 1051–1064 (1984) 6743742 10.1016/S0006-3495(84)84252-6 PMC1434996

[43] C. Zhu, Kinetics and mechanics of cell adhesion. J. Biomech. **33**(1), 23–33 (2000) 10609515 10.1016/s0021-9290(99)00163-3

[44] G. von Winckel, Legendre-Gauss Quadrature Weights and Nodes,MATLAB Central File Exchange. https://www.mathworks.com/matlabcentral/fileexchange/4540-legendre-gauss-quadrature-weights-and-nodes Accessed 2021-04-15 (2021)

[45] R.J. Moon, A. Martini, J. Nairn, J. Simonsen, J. Youngblood, Cellulose nanomaterials review: structure, properties and nanocomposites. Chem. Soc. Rev. **40**(7), 3941–3994 (2011) 21566801 10.1039/c0cs00108b

[46] W. Gindl, T. Schöberl, The significance of the elastic modulus of wood cell walls obtained from nanoindentation measurements. Compos. A Appl. Sci. Manuf. **35**(11), 1345–1349 (2004)

[47] J. Gassan, A. Chate, A.K. Bledzki, Calculation of elastic properties of natural fibers. J. Mater. Sci. **36**(15), 3715–3720 (2001)

[48] S. Iwamoto, W. Kai, A. Isogai, T. Iwata, Elastic modulus of single cellulose microfibrils from tunicate measured by atomic force microscopy. Biomacromol **10**(9), 2571–2576 (2009) 10.1021/bm900520n 19645441

[49] S. Huang, M. Makarem, S.N. Kiemle, H. Hamedi, M. Sau, D.J. Cosgrove, S.H. Kim, Inhomogeneity of cellulose microfibril assembly in plant cell walls revealed with sum frequency generation microscopy. J. Phys. Chem. B **122**(19), 5006–5019 (2018) 29697980 10.1021/acs.jpcb.8b01537

[50] L. Wilson, F. Deligey, T. Wang, D.J. Cosgrove, Saccharide analysis of onion outer epidermal walls. bioRxiv (2021) 10.1186/s13068-021-01923-z PMC7962260 33722273

[51] D. Ye, S. Rongpipi, S.N. Kiemle, W.J. Barnes, A.M. Chaves, C. Zhu, V.A. Norman, A. Liebman-Peláez, A. Hexemer, M.F. Toney et al., Preferred crystallographic orientation of cellulose in plant primary cell walls. Nat. Commun. **11**(1), 1–10 (2020) 32948753 10.1038/s41467-020-18449-x PMC7501228

[52] J.A. Fozard, M. Lucas, J.R. King, O.E. Jensen, Vertex-element models for anisotropic growth of elongated plant organs. Front. Plant Sci. **4**, 233 (2013). 10.3389/fpls.2013.00233 23847638 10.3389/fpls.2013.00233 PMC3706750

[53] R. Dyson, O. Jensen, A fibre-reinforced fluid model of anisotropic plant cell growth. J. Fluid Mech. **655**, 472–503 (2010)

[54] M. Ptashnyk, B. Seguin, The impact of microfibril orientations on the biomechanics of plant cell walls and tissues. Bull. Math. Biol. **78**(11), 2135–2164 (2016) 27761699 10.1007/s11538-016-0207-8 PMC5090020

[55] W. Guo, J. Zhao, X. Li, L. Qin, X. Yan, H. Liao, A soybean -expansin gene gmexpb2 intrinsically involved in root system architecture responses to abiotic stresses. Plant J. **66**(3), 541–552 (2011) 21261763 10.1111/j.1365-313X.2011.04511.x

[56] H. Zou, Y. Wenwen, G. Zang, Z. Kang, Z. Zhang, J. Huang, G. Wang, Osexpb2, a -expansin gene, is involved in rice root system architecture. Mol. Breeding **35**, 1–14 (2015)

[57] P. Marowa, A. Ding, Y. Kong, Expansins: roles in plant growth and potential applications in crop improvement. Plant Cell Rep. **35**, 949–965 (2016) 10.1007/s00299-016-1948-4 PMC4833835 26888755

[58] E. Miedes, I. Zarra, T. Hoson, K. Herbers, U. Sonnewald, E. Lorences, Xyloglucan endotransglucosylase and cell wall extensibility. J. Plant Physiol. **168**(3), 196–203 (2011) 20828871 10.1016/j.jplph.2010.06.029

[59] N. Kaewthai, D. Gendre, J.M. Eklöf, F.M. Ibatullin, I. Ezcurra, R.P. Bhalerao, H. Brumer, Group iii-a xth genes of arabidopsis encode predominant xyloglucan endohydrolases that are dispensable for normal growth. Plant Physiol. **161**(1), 440–454 (2013) 23104861 10.1104/pp.112.207308 PMC3532273

[60] H. Kuki, R. Yokoyama, T. Kuroha, K. Nishitani, Xyloglucan is not essential for the formation and integrity of the cellulose network in the primary cell wall regenerated from arabidopsis protoplasts. Plants **9**(5), 629 (2020) 32423049 10.3390/plants9050629 PMC7285283

[61] S.L. Sridhar, J.K. Ortega, F.J. Vernerey, A statistical model of expansive growth in plant and fungal cells: The case of phycomyces. Biophys. J . **115**(12), 2428–2442 (2018) 30514633 10.1016/j.bpj.2018.11.014 PMC6302256

[62] E.F. Crowell, H. Timpano, T. Desprez, T. Franssen-Verheijen, A.-M. Emons, H. Höfte, S. Vernhettes, Differential regulation of cellulose orientation at the inner and outer face of epidermal cells in the arabidopsis hypocotyl. Plant Cell **23**(7), 2592–2605 (2011) 21742992 10.1105/tpc.111.087338 PMC3226210

